# OSCAR: Optimal subset cardinality regression using the L0-pseudonorm with applications to prognostic modelling of prostate cancer

**DOI:** 10.1371/journal.pcbi.1010333

**Published:** 2023-03-10

**Authors:** Anni S. Halkola, Kaisa Joki, Tuomas Mirtti, Marko M. Mäkelä, Tero Aittokallio, Teemu D. Laajala

**Affiliations:** 1 Department of Mathematics and Statistics, University of Turku, Turku, Finland; 2 Research Program in Systems Oncology, Faculty of Medicine, University of Helsinki, Helsinki, Finland; 3 Department of Pathology, Diagnostic Center, Helsinki University Hospital, Helsinki, Finland; 4 Department of Biomedical Engineering, School of Medicine, Emory University, Atlanta, Georgia, United States of America; 5 Institute for Molecular Medicine Finland (FIMM), HiLIFE, University of Helsinki, Helsinki, Finland; 6 Institute for Cancer Research, Oslo University Hospital, Oslo, Norway; 7 Oslo Centre for Biostatistics and Epidemiology (OCBE), University of Oslo, Oslo, Norway; 8 Department of Pharmacology, University of Colorado Anschutz Medical Campus, Aurora, Colorado, United States of America; Academy of Mathematics and Systems Science, Chinese Academy of Science, CHINA

## Abstract

In many real-world applications, such as those based on electronic health records, prognostic prediction of patient survival is based on heterogeneous sets of clinical laboratory measurements. To address the trade-off between the predictive accuracy of a prognostic model and the costs related to its clinical implementation, we propose an optimized *L*_0_-pseudonorm approach to learn sparse solutions in multivariable regression. The model sparsity is maintained by restricting the number of nonzero coefficients in the model with a cardinality constraint, which makes the optimization problem NP-hard. In addition, we generalize the cardinality constraint for grouped feature selection, which makes it possible to identify key sets of predictors that may be measured together in a kit in clinical practice. We demonstrate the operation of our cardinality constraint-based feature subset selection method, named OSCAR, in the context of prognostic prediction of prostate cancer patients, where it enables one to determine the key explanatory predictors at different levels of model sparsity. We further explore how the model sparsity affects the model accuracy and implementation cost. Lastly, we demonstrate generalization of the presented methodology to high-dimensional transcriptomics data.

## Author summary

Feature subset selection has become a crucial part of building biomedical models, due to the abundance of available predictors in many applications, yet there remains an uncertainty of their importance and generalization ability. Regularized regression methods have become popular approaches to tackle this challenge by balancing the model goodness-of-fit against the increasing complexity of the model in terms of coefficients that deviate from zero. Regularization norms are pivotal in formulating the model complexity, and currently *L*_1_-norm (LASSO), *L*_2_-norm (Ridge Regression) and their hybrid (Elastic Net) dominate the field. In this paper, we present a novel methodology that is based on the *L*_0_-pseudonorm, also known as the best subset selection, which has largely gone overlooked due to its challenging discrete nature. Our methodology makes use of a continuous transformation of the discrete optimization problem, and provides effective solvers implemented in a user friendly R software package. We exemplify the use of *oscar*-package in the context of prostate cancer prognostic prediction using both real-world hospital registry and clinical cohort data. By benchmarking the methodology against existing regularization methods, we illustrate the advantages of the *L*_0_-pseudonorm for better clinical applicability, selection of grouped features, and demonstrate its applicability in high-dimensional transcriptomics datasets.

This is a *PLOS Computational Biology* Methods paper.

## Introduction

Current cancer incidence is more than 19 million new cases per year and rapidly rising globally [1]. Despite the successful development of medical treatments that have decreased the mortality of cancer patients, cancer still remains one of the most common causes of death, thus leading to dire need for more precise and prognostic insights into patient care. Prognostic prediction is fundamental in patient management, since it enables the assessment of prognosis in diagnostic phase and prediction of the disease course for an individual patient after treatment or disease relapse. Predicting the risk of cancer recurrence or death, based on the individual patient characteristics and laboratory measurements, helps to understand which patients would benefit from a standard treatment and which are better assigned to a palliative care or treated with alternative therapy regimens. In clinical practice, survival prediction is typically carried out using laboratory tests, which are many times numerous and thus expensive. From a health economics point of view, prognostic modelling should be both accurate and cost-effective, and the prognostic models should not become too complex to enable their clinical implementation. In this particular aspect, feature selection strategies, such as regularization in regression modelling, play a key role.

Prostate cancer is one of the most common cancers diagnosed in men and among the top causes of cancer mortality [1]. Although the prognosis of prostate cancer is generally good, a considerable number of patients either have a metastasized disease at the time of diagnosis or they develop a potentially lethal recurrent disease after the initial treatment. Prostate-specific antigen (PSA) is currently considered as the default marker of disease progression in the disease and treatment monitoring. However, when prostate cancer develops into a hormonal treatment independent state (i.e. castration resistant prostate cancer), more rigorous testing using additional markers is needed for more accurate patient stratification [2]. Given the high prevalence of prostate cancer globally, it is not trivial how to consider the costs of the more extensive testing during follow-up, further increasing the need for cost-effective risk prediction strategies.

Risk classification models for prostate cancer are traditionally applied either at the diagnostic phase or primary treatment phase. Most of the current prognostic models contain Gleason score, which is considered the most significant factor for early disease course estimation [3]. In contrast, our objective here was to make prognostic prediction of patients who have already developed metastatic castration-resistant prostate cancer, and therefore seek to investigate prognostic features beyond Gleason score. Regularized Cox regression models have been a popular choice for such prognostic modelling purposes [4–8]. For example, in the DREAM 9.5 Prostate Cancer Prediction Challenge [6], our top-performing model was based on an ensemble of regularized models with Cox regression [9].

Our prognostic modelling framework for prostate cancer is based on the Cox’s proportional hazards model [5, 10], which is extended by introducing a novel feature selection regularization strategy. More specifically, we use a cardinality constraint implemented by the *L*_0_-pseudonorm to restrict the number of nonzero coefficients. Such cardinality constraint complicates the optimization, since this constraint is discontinuous and nonconvex, which makes the problem NP-hard (Nondeterministic Polynomial hard) [11]. Previous modelling approaches with *L*_0_-implementations have been developed for generalized linear models, such as linear and logistic regression [12–14], but they do not offer solutions for the Cox model essential for prognostic predictions. For example, the SDAR method in [14] relies on Karush-Kuhn-Tucker optimality conditions for the *L*_0_-penalized least squares solutions, thus being applicable only for linear regression models. To the best of our knowledge, there is only one *L*_0_-implementing Cox’s proportional hazards model, the augmented penalized minimization-*L*_0_ (APM-*L*_0_) [15], which approximates the *L*_0_ approach, and iterates between a coordinate descent-based convex regularized regression and a simple hard-thresholding estimation. However, since the APM-*L*_0_ method incorporates also *L*_1_- and *L*_2_-norms, it is unclear whether or not it performs fundamentally differently compared, for example, with LASSO.

Our implementation differs from the APM-*L*_0_ approach. First, we rewrite the cardinality constraint with its exact DC (Difference of two Convex functions) representation, after which the constraint is added to the objective function utilizing a penalty function approach [16]. This leads to a continuous nonsmooth (i.e. not necessarily continuously differentiable) objective function, whereas the nonconvexity remains even after the transformation. In our methodology, the optimization is done with two sophisticated solvers: the double bundle method (DBDC) [17, 18] for nonsmooth DC optimization, and the limited memory bundle method (LMBM) [19, 20] for nonsmooth large-scale optimization. Both solvers are capable of handling the exact DC representation of the cardinality-constrained problem after it has been transformed into a penalty function form. In addition to the advanced optimization methods and inclusion of the cardinality constraint, we further generalize the cardinality constraint to also control the number of used kits that group together multiple predictors that come with the same cost in clinical practice. Instead of a single measurement, in practice, many features are often measured together as kits (such as complete blood count). In our methodology, such kit structure can be included in the model, thus enabling the selection of relevant predictor subsets instead of single predictors only.

In this work, we present a new *L*_0_ regularization method OSCAR (Optimal Subset CArdinality Regression) and exemplify it with Cox’s proportional hazards model in prognostic prediction of prostate cancer. The OSCAR method is tested in multiple data cohorts: TYKS (real-world hospital registry data) [8], VENICE, MAINSAIL and ASCENT (randomized clinical trials) [6], and TCGA, Taylor et al. and Sun et al. (high-dimensional transcriptomics datasets) [21–23]. We use bootstrap (BS) and cross-validation (CV) analyses to ensure robustness and generalization ability of the model. For TYKS, VENICE, MAINSAIL, and ASCENT, the model prediction accuracy is also investigated alongside the corresponding predictor costs; this helps to identify models that are also cost-effective (i.e. max accuracy, min cost). Combining these two objectives leads to a multi-objective optimization problem. We note that the process of fitting the Cox’s proportional hazards model (i.e. accuracy) for all the required cardinalities is one way to obtain an approximation of the Pareto-front [24] in this multi-objective problem. These Pareto-fronts can then be provided for the end-users for decision making. Finally, the models selected based on the Pareto-fronts are further tested in validation cohorts independent from the training datasets and pre-selected before model fitting. We compare the OSCAR results to the traditional LASSO [4], *L*_0_-augmented APM-*L*_0_ [15], smoothly clipped absolute deviation (SCAD) penalized regression [25], and greedy forward selection (Greedy FS).

## Materials and methods

### OSCAR method

**Fig 1:**
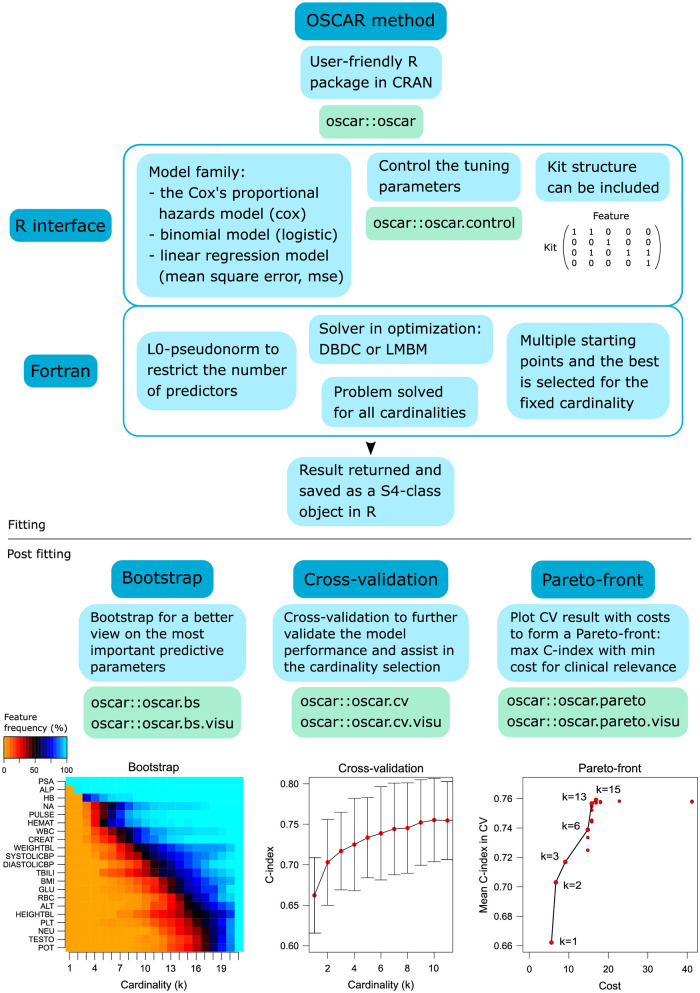
Schematic illustration of the OSCAR method.

In the OSCAR method, we refer to cardinality as the number of predictors or groups of predictors (i.e. kits) in the model. Schematic illustration of the OSCAR methodology is presented in Fig 1. In addition to survival prediction, the OSCAR method implements binomial model for logistic regression problems and linear regression with mean square error (see e.g. [26]).

#### Cox’s proportional hazards model

Our modelling interest is mainly in the patient survival prediction, where we investigate the relationship between patient features (see subsection “Prostate cancer data for testing”) and survival time (overall survival or progression free survival). In the general form, this type of data can be stated as a set

$$\begin{array}{c} A=\{\left(x_{i},\;y_{i},\;\delta_{i}\right)\in R^{p}\times R_{+}\times \left\{0,1\right\}\;|\;\;i=1,\ldots ,n\}, \end{array}$$ (1)

where *n* is the number of observations, $x_{i}\in R^{p}$ is the vector of *p* features, $y_{i}\in R_{+}$ is the observed time and *δ*_*i*_ ∈ {0, 1} is the label (value 1 indicates an event, typically a failure, and value 0 right-censoring). In addition, we let *t*_1_ < *t*_2_ < … < *t*_*m*_ be increasing list of *m* unique failure times, and *D*_*i*_ be the set of indices of observations failing at time *t*_*i*_, meaning that ties are also allowed to happen.

Survival prediction is traditionally carried out using Cox’s proportional hazards model [10]. The *hazard* for the patient *i* at time *t* is given with the formula

$$\begin{array}{c} h_{i}\left(t\right)=h_{0}\left(t\right)e^{x_{i}^{⊤}\beta }, \end{array}$$

where *h*_0_(*t*) is a shared baseline hazard and $\beta \in R^{p}$ is an unknown coefficient vector. Our aim is to estimate this vector ***β*** by maximizing the Breslow approximation of the *partial likelihood* (see [27]). In the following, we denote by $\bar{\beta }$ the solution yielding the maximum value for the likelihood.

Instead of maximizing the partial likelihood directly, it is also possible to maximize the scaled log partial likelihood, since this leads to an equivalent solution [5]. This modification gives the *scaled log partial likelihood* of the form

$$\begin{array}{c} l\left(\beta \right)=\frac{2}{n}\sum_{i=1}^{m}\{\sum_{j\in D_{i}}x_{j}^{⊤}\beta -d_{i}\;\text{ln}(\sum_{j\in R_{i}}e^{x_{j}^{⊤}\beta })\}, \end{array}$$ (2)

where *R*_*i*_ = {*j* : *y*_*j*_ ≥ *t*_*i*_} is the set of indices at risk at time *t*_*i*_ and *d*_*i*_ = |*D*_*i*_| is the number of failures at time *t*_*i*_. The function −*l* is convex, since it is a sum of linear and log-sum-exp functions [28]. Therefore, instead of maximizing the concave function *l*, it is equivalent to minimize the convex function −*l*. In the following, we concentrate on solving the minimization problem

$$\begin{array}{c} \underset{\beta \in R^{p}}{\text{min}}\;-l\left(\beta \right), \end{array}$$ (3)

whose solution $\bar{\beta }$ also maximizes (2).

#### Restricting the number of model features

In many real-world applications, it is beneficial to have sparse solutions for the partial likelihood function. To favor sparse solutions, a regularization term is typically added to the optimization problem. For example, the elastic net penalization is used in [5] by combining *L*_1_- and *L*_2_-norms. In particular, approaches relying on the *L*_1_-norm ensure sparsity to a certain extent.

In our approach, the sparsity of the solution is obtained by using the cardinality constraint to restrict the number of nonzero coefficients in the vector ***β***. The strength of this approach is that it provides us an effective tool to seek solutions with a predetermined model complexity. Instead of considering each feature separately, we may also want to group some features together, if they are always measured together (i.e. they belong to the same measurement kit). Therefore, we also generalize the cardinality constraint-based feature subset selection to a case where we restrict the number of selected kits (see section 1 in S1 Text for restricting the number of selected kits, instead of single features).

For any vector $\beta \in R^{p}$, the *L*_0_-*pseudonorm* ‖***β***‖_0_ calculates the number of nonzero components. However, it is worth noting that the *L*_0_-pseudonorm is not a proper norm since it is not homogeneous [29], thus the name pseudonorm. In addition, this pseudonorm is discontinuous and nonconvex, making the optimization problem much more challenging [16, 30].

In the problem (3), sparsity can be achieved by fixing the number of nonzero coefficients *K* ∈ {1, …, *p*} and adding a *cardinality constraint* ‖***β***‖_0_ ≤ *K*. This results in the following *cardinality-constrained problem*

$$\begin{array}{c} \left\{ \begin{array}{cc} \underset{\beta \in R^{p}}{\text{min}}\; & -l(\beta )\\ s.t. & {∥\beta ∥}_{0}\leq K. \end{array}\right. \end{array}$$ (4)

It is known that this problem is difficult to solve due to the combinatorial nature of the constraint, which is also discontinuous. To overcome the discontinuity, we use the approach presented in [16], which utilizes the largest-*k* norm to obtain an exact continuous representation of the constraint.

The *largest-k norm* of a vector $\beta \in R^{p}$ is the sum of the *k* largest absolute value elements:

$$\begin{array}{c} {∥|\beta |∥}_{\left[k\right]}\;≔\;|\beta_{\left(1\right)}\left|+\right|\beta_{\left(2\right)}\left|+\ldots +\right|\beta_{\left(k\right)}|, \end{array}$$

where *β*_(*i*)_ is the element whose absolute value is the *i*-th largest among the *p* elements of ***β***. The largest-*k* norm is a proper norm. In addition, it is convex and the constraint ‖***β***‖_0_ ≤ *K* is equivalent to the constraint ${∥\beta ∥}_{1}-{∥\;|\beta ∥\;|}_{\left[K\right]}=0$ [16, 30], where ${∥\beta ∥}_{1}\;≔\;|\beta_{1}\left|+\right|\beta_{2}\left|+\ldots +\right|\beta_{p}|={∥\;|\beta ∥\;|}_{\left[p\right]}$. Thus, the problem (4) can be rewritten as

$$\begin{array}{c} \left\{ \begin{array}{cc} \underset{\beta \in R^{p}}{\text{min}}\; & -l(\beta )\\ s.t. & {∥\beta ∥}_{1}-{∥\;|\beta ∥\;|}_{\left[K\right]}=0 \end{array}\right. \end{array}$$ (5)

and we have a continuous constraint instead of a discontinuous one. Note that both problems (4) and (5) have exactly the same feasible set. However, the combinatorial structure of the cardinality constraint causes the continuous constraint to be nonconvex. For this reason, the problem (5) may have multiple local solutions and identifying a global or near global solution requires a sophisticated optimizer.

Another disadvantage of the problem (5) is that we still have a constraint. Similarly to [16], we can utilize the penalty function approach [31, 32] to rewrite the constrained problem (5) as an unconstrained one

$$\begin{array}{c} \underset{\beta \in R^{p}}{\text{min}}\;f\left(\beta \right)=-l\left(\beta \right)+\rho ({∥\beta ∥}_{1}-{∥\;|\beta ∥\;|}_{\left[K\right]}\;), \end{array}$$ (6)

where *ρ* > 0 is a positive penalization parameter. In this reformulation, we are balancing between feasibility and optimality. By selecting a too small value for the parameter *ρ*, we do not obtain a feasible solution for the original problem (5). However, by selecting a suitably large value for *ρ*, we have a heavy cost for cardinality constraint violation and end up with a feasible solution. Note that the parameter *ρ* should not be too large since otherwise the penalty term dominates the objective function and in practice we do not obtain an optimal solution for the objective of the constrained problem (5). For this reason, as is typical for penalty function methods, we need to solve the problem (6) sequentially for a series of increasing values of the parameter *ρ* until a suitable parameter value is reached that forces the original constraint in (4) to hold. In practice, this search is done by using a *ρ* value-grid.

One major benefit of the formulation in (6) is that, although its objective *f* is nonconvex and nonsmooth, it is a *DC function* (Difference of two Convex functions). This means that *f* can be represented in the form *f* = *f*^1^ − *f*^2^ with two convex functions *f*^1^ and *f*^2^. This way we can better control the nonconvexity than in the general case. In addition, these convex functions can be selected, for example, as

$$\begin{array}{c} f^{1}\left(\beta \right)=-l\left(\beta \right)+\rho {∥\beta ∥}_{1}\;\;\text{and}\;\;f^{2}\left(\beta \right)=\rho {∥\;|\beta ∥\;|}_{\left[K\right]}. \end{array}$$

Another interesting aspect of the penalized reformulation (6) is that it can be seen as a modification of the *L*_1_-norm based penalization since the only difference is the largest-*k* norm term $-\rho {∥\;|\beta ∥\;|}_{\left[K\right]}$. Note that this is the term restricting and controlling the upper bound for the number of nonzero features in the problem.

#### OSCAR algorithm

In this section, we introduce the new algorithm OSCAR (Optimal Subset CArdinality Regression) to solve the cardinality-constrained problem formulated in (4). Since the considered problem is nonconvex, it is well-known that the determination of a global solution is a challenging task, since there may be many local optima and we lack easily verified conditions guaranteeing the global optimality. Due to this, the goal of our new local optimization framework is to find good enough solutions which are close to the global optima. To achieve this goal, our method combines first time the penalty function approach and the double bundle method (DBDC) [17, 18] for DC optimization together with an incremental type of an approach to solve the original problem.

OSCAR methodology is designed so, that it does not depend on the specific optimization method, provided the method is capable of handling both nonsmoothness and nonconvexity. Therefore, our method generalizes beyond DBDC, although it is offered as the default choice for small-scale problems with *p* < 100 features. Due to this, we have also incorporated into the R-package of OSCAR the possibility to use the limited memory bundle method (LMBM) [19, 20]. LMBM is designed for general nonconvex nonsmooth optimization problems, with the drawback, that it does not benefit from the DC structure of the objective. The most important feature of LMBM is that it scales towards large-scale problems and thus it is given as a default solver for the cases where *p* ≥ 100. In addition to boosting calculations with high-dimensional data, we introduce an acceleration procedure that utilizes a one-dimensional subproblem and a reduced-sized original problem (4) (see section 2 in S1 Text for the acceleration procedure for high-dimensional data).

As described above, the first step of OSCAR is to use the penalty function approach to transform the rewritten constrained problem (5) to an unconstrained one. Since the objective of the unconstrained problem (6) is DC, we can solve it by utilizing the DBDC method for DC optimization. This enables us to take advantage of the DC structure, since the selected bundle method constructs a nonconvex DC cutting plane model (i.e. an approximation of the objective function, which incorporates both the convex and the concave behavior of the problem). Another option to solve the problem (6) is LMBM as described above.

However, since DBDC and LMBM are local optimizers, the quality of solutions for a nonconvex problem strongly depends on the choice of starting points. For this reason, the algorithm OSCAR combines the DBDC and LMBM methods with an incremental type of an approach to generate multiple starting points with higher likelihood of leading to most promising parts of the search space. The idea in our incremental approach is to start with solving the cardinality-constrained problem, where only a single predictor (or kit) is allowed to be used initially, and then to increase the number of predictors (or kits) one at a time until the maximal number of predictors is achieved. In particular, we utilize the solution of the cardinality-constrained problem with *i* − 1 predictors to identify promising starting points to the next cardinality-constrained problem with *i* predictors. Since this type of incremental forward selection process may end up in a local optimum, we alleviate this via the use of multiple starting points to obtain multiple solution candidates for the problem with *i* predictors (so-called multi-start global optimization).

**Algorithm 1:** OSCAR

**Input**: The values of features ***x***_*i*_, the survival times *y*_*i*_, the labels *δ*_*i*_ ∈ {0, 1} and the maximal number of predictors *K*_*max*_ ∈ {1, 2, …, *p*} until which the cardinality-constrained problem is solved.

**Output**: For *i* = 1, …, *K*_*max*_, gives the solution $\beta_{i}^{*}$ for the cardinality-constrained problem with *i* predictors.

**Step 0**: (*Initialization*) Solve the convex problem (3) with DBDC or LMBM and denote the solution by $\bar{\beta }$. Set $\beta_{0}^{*}=0$ and *i* = 1.

**Step 1**: (*Starting points*) For the cardinality-constrained problem with *i* predictors, initialize the set of starting points *S*_*i*_ = ∅. For *j* = 1, …, *p* construct the point $\beta_{0}^{j}$ with the formula

$$\begin{array}{c} \beta_{0,l}^{j}=\{\begin{array}{cc} \beta_{i-1,l}^{*}\; & \;\text{for}\;l=1,\ldots ,p\;\text{and}\;l\neq j\\ {\bar{\beta }}_{l}\; & \;\text{for}\;l=j \end{array} \end{array}$$

and if $∥\beta_{0}^{j}{∥}_{0}>i-1$ then add the point to the set *S*_*i*_.

**Step 2**: (*Penalty function problem*) Do the following steps A–C for all $\beta_{0}^{j}\in S_{i}$ to obtain solutions $\beta_{i,j}^{*}$

 **Step A**: Select a positive initial value for the penalization parameter *ρ*.

 **Step B**: Solve the problem (6) with the DBDC or LMBM method starting from $\beta_{0}^{j}$ and denote the solution with ${\hat{\beta }}_{j}$.

 **Step C**: If $∥{\hat{\beta }}_{j}{∥}_{0}=i$, then set $\beta_{i,j}^{*}={\hat{\beta }}_{j}$. Otherwise increase the value of the penalization parameter *ρ* and go to Step B.

**Step 3**: (*Solution*) Select the best solution $\beta_{i}^{*}$ for the cardinality-constrained problem (5) with *i* predictors using the formula

$$\begin{array}{c} \beta_{i}^{*}=\underset{j}{\text{arg}\;\text{min}}\;\left\{\;-l\left(\beta_{i,j}^{*}\right)\;\right\}. \end{array}$$

 Update *i* = *i* + 1. If *i* ≤ *K*_*max*_, then go to Step 1. Otherwise go to Step 4.

**Step 4**: Return $\beta_{i}^{*}$ for all *i* = 1, …, *K*_*max*_.

The OSCAR method is presented in Algorithm 1 for the case where each predictor is considered separately. See section 1 in S1 Text for modifications needed with a kit structure of grouped features. As an input, one needs to give the maximal number of predictors *K*_*max*_, defining how many predictors maximally can be selected in the densest cardinality-constrained problem. As an output, the method provides incrementally a solution to each cardinality-constrained problem with *i* predictors for *i* = 1, …, *K*_*max*_ and, thus, one obtains as a by-product a solution also for each cardinality-constrained problem with a smaller number of used predictors (or kits). This means that one can control how many different sparse solutions are generated. Naturally, it is also possible to select *K*_*max*_ = *p*, in which case the problem (5) is solved for all the possible numbers of predictors.

In Step 1 of Algorithm 1, starting points are generated by varying the previous solution $\beta_{i-1}^{*}$ with the best solution $\bar{\beta }$ of the scaled log partial likelihood obtained without any regularization. More specifically, in a starting point $\beta_{0}^{j}$ the base is $\beta_{i-1}^{*}$, and then one can simply substitute predictor *j* with the corresponding value in $\bar{\beta }$. In this way, one can easily vary the previous solution but still maintain its main predictors. Note also that each starting point with *i* − 1 predictors is omitted and we only keep the starting points with *i* predictors.

In Step 2B of Algorithm 1, we always use the original starting point. The reason for this is that if the parameter *ρ* is too small, then we may end up with a solution where nearly all the coefficients are nonzero, and therefore, lose the information provided by the original starting point. To avoid such solutions, we do not change the starting point, but instead update the parameter *ρ* until we obtain a solution with the acceptable number of predictors (or kits). This guarantees that the obtained solution does not diverge too much from the previous solution and maintains its best predictors. In addition, this way the method does not become too sensitive to the selection of *ρ*, since too small values of *ρ* are basically omitted.

### Prostate cancer data for testing

For testing the new algorithm for survival prediction, we used one prostate cancer cohort from real-world hospital registry data, three prostate cancer cohorts from randomized clinical trials (see Table A in S1 Text), and three publicly available high-dimensional transcriptomics datasets with biochemical recurrence information. The patient-specific features were also considered by the clinical examination groups (kits), in which they are measured in clinical practice. Prices for the examinations were obtained from the standard laboratory test costs of the Helsinki University Hospital (Finland). The real prices were converted to costs relative to PSA, which was given a reference value of 100. One feature (blood urea nitrogen) without a known cost was ignored. The features are shown in Table 1, along with abbreviations as well as the kit structures and standardized prices.

**Table 1:** Data features for the clinical cohort variables.

Abbreviation	Meaning	Unit	Kit	Price
AGEGRP	Age group (three groups)	-	Routine measurements	0
BMI	Body mass index	kg/m^2^		
DIASTOLICBP	Diastolic blood pressure	mmHg		
HEIGHTBL	Height	cm		
PULSE	Pulse	bpm		
SYSTOLICBP	Systolic blood pressure	mmHg		
WEIGHTBL	Weight	kg		
HB	Hemoglobin	g/dl	B-PVKT	40
HEMAT	Hematocrit	%		
PLT	Platelets	E9/l		
RBC	Red blood cells	E12/l		
WBC	White blood cells	E9/l log		
NEU	Neutrophils	E9/l log	TKD	50
POT	Potassium	mmol/l		
ALP	Alkaline phosphatase	U/l log	P-AFOS	20
ALT	Alanine aminotransferase	U/l log	P-Alat	20
AST	Aspartate aminotransferase	U/l log	P-AsaT	20
CA	Calcium	mmol/l	P-Ca	20
CREAT	Creatinine	umol/l log	P-Krea	20
LDH	Lactate dehydrogenase	U/l log	P-LD	20
PSA	Prostate-specific antigen	ng/ml log	P-PSA	100
TBILI	Bilirubin	umol/l log	P-Bil	20
TESTO	Testosterone	nmol/l log	S-Testo	330
NA	Sodium	mmol/l	cB-Het-Ion	100
MG	Magnesium	mmol/l log	P-Mg	20
PHOS	Phosphorus	mmol/l log	P-Pi	20
ALB	Albumin	g/l	P-ALB	20
TPRO	Total protein	g/l	S-Prot	20
LYM	Lymphocytes	E9/l log	B-Lymf	90
CCRC	Calculated creatinine clearance	ml/min log	Pt-GFReEPI	20
GLU	Glucose	mmol/l log	Gluk	20

Abbreviations, explanations and units for features used in the analyses, as well as kit structures and corresponding prices. Prices were standardized so that PSA has a price of 100.

#### Real-world hospital registry data

**Fig 2:**
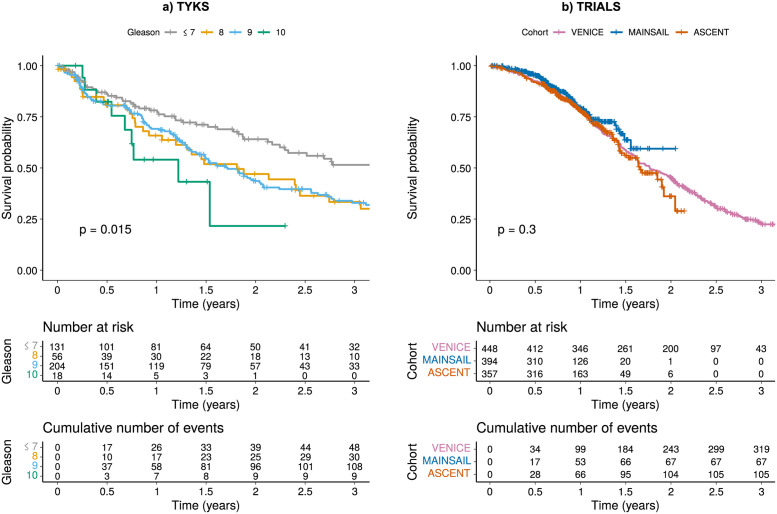
**Survival curves:** a) Kaplan-Meier survival probability for the TYKS patient cohort based on the Gleason scores. b) Kaplan-Meier survival probability for the three clinical trial cohorts: VENICE, MAINSAIL, and ASCENT.

The real-world hospital registry data were collected from the advanced prostate cancer patients treated at the Turku University Hospital (TYKS, Finland). Patients with castration resistance were selected and data processed as in [9]. Furthermore, only patients with diagnosis of castration resistance dated in 2010 or later were selected, due to the higher sparsity of data in the previous years. In addition, patients with zero or negative survival time or no measurement data were discarded. A total of 195 patients were set aside to be used as an independent validation dataset to evaluate the generalization capability of the model, and to assess the risk of over-fitting to the training dataset. We further eliminated features with over 50% of missing values. The remaining missing data were imputed using median values calculated in the training dataset (*n* = 590). Median imputation has been previously tested and found adequate [8, 9]. One outlier measurement of systolic blood pressure (>12 000 mmHg) was changed into missing before imputation. Patient characteristics for the training data are presented in Table A in S1 Text, and the survival curves in the TYKS cohort with respect to the Gleason scores are shown in Fig 2a. The survival curves behaved as expected, with lowest survival on the highest Gleason scores and highest survival on the lowest Gleason scores. Since between-feature correlations may affect the feature selection process, we investigated these across the available features as shown in Fig A in S1 Text.

#### Randomized clinical trial data

The randomized clinical trial data included in the analyses were previously constructed in the DREAM 9.5 competition (the Prostate Cancer Challenge, PCC-DREAM), hosted by Project Data Sphere (https://www.projectdatasphere.org/). These data come from three prostate cancer patient cohorts (MAINSAIL, VENICE, and ASCENT) with metastatic castration-resistant prostate cancer [33–35]. From each cohort, a random set of patients was separated as a validation dataset (*n* = 132, *n* = 150 and *n* = 119 for MAINSAIL, VENICE and ASCENT, respectively). Features with over 50% of missing values were eliminated. Missing values in each cohort were imputed separately using median values calculated from the corresponding training datasets (*n* = 394, *n* = 448 and *n* = 357 for MAINSAIL, VENICE and ASCENT, respectively). Patient characteristics are presented in Table A in S1 Text, and the survival curves per cohort are shown in Fig 2b. The survival curves start similarly, however, the MAINSAIL and ASCENT cohort have a shorter follow-up time. The overall survival trend was similar to the TYKS real-world cohort (Fig 2a). We also present the correlations between features of the clinical trial cohorts in Fig A in S1 Text.

#### High-dimensional transcriptomic datasets for prostate cancer recurrence

TCGA [21], Taylor et al. [22], and Sun et al. [23] datasets were obtained as described in section 6.1 in S1 Text using the *curatedPCaData*-package. Only primary tumors were used for model training and validation, with the end-point being disease recurrence. In TCGA and Taylor et al., recurrence information was available along with follow-up times. However, in Sun et al., recurrence was a binarized outcome without follow-up times. Therefore, C-index was used as the performance metric in TCGA and Taylor et al., while ROC-AUC was used in Sun et al. for the performance metric for evaluating the ability distinguish between recurring and non-recurring tumors.

For analysis of computational cost, the full TCGA (original *n* = 404, *p* = 19 658, with 76 observed recurrence events) and Taylor et al. (*n* = 131, *p* = 17 410, with 27 observed recurrence events) datasets were used while examining how the computational burden increased as a function of data dimensionality. Since Sun et al. (*n* = 79, *p* = 12 783, with 39 recurrent and 40 non-recurrent sample) provided only the recurrence as a binarized outcome, it was not included in the computational benchmarking of the Cox model fitting with OSCAR, but it was included in the validation of the generalization ability of identified models when using OSCAR and the other benchmarking methods.

For model validation, the z-score transformation was applied across genes to harmonize the datasets. Furthermore, genes with less than 50% unique values across the patient samples were omitted to avoid redundant variables (i.e. genes with little or no variability, and hence having a limited predictive value). Lastly, in order to make the identified genes as comparable as possible across the datasets, genes were subset to common gene names. This resulted in a validation data dimensionality of *p* = 10 253 across the three transcriptomics datasets.

## Results

We first investigated the performance of our OSCAR method in four clinical patient cohort-based prostate cancer datasets, which portrait two very distinct archetypes of biomedical data. First, we applied the method to the advanced prostate cancer cohort obtained from Turku university hospital (TYKS), representing a highly heterogeneous real-world hospital registry patient cohort. Second, we applied the method to three prostate cancer cohorts obtained from randomized clinical trials, which had been part of a DREAM prostate cancer modelling challenge and had been homogenized previously by the challenge organizers. Lastly, to investigate the method performance in high-dimensional transcriptomic data from prostate cancer patients, we used three additional gene expression-based datasets (TCGA, Taylor et al., and Sun et al.), which all have data dimensionality above *p* > 10 000 and disease recurrence as the prediction end-point.

The predictive performance was evaluated with Harrel’s concordance index (C-index) [36] when the patient follow-up information was available. C-index is commonly used in survival analysis as it compares the order of predicted risks to the order of observed survival times [37–39]. In Sun et al. dataset, where recurrence follow-up times were not available, the commonly used Receiver Operator Characteristic Area Under Curve (ROC-AUC) was used as the performance metric.

To benchmark OSCAR performance, we compared its results to a widely used method LASSO [4], which utilizes *L*_1_-regularization. We included another *L*_0_-pseudonorm based method APM-*L*_0_ [15], which was chosen based on literature search for *L*_0_-related methods capable of performing survival analysis. We further compared OSCAR to a SCAD-penalized regression method [25]. Furthermore, the greedy forward selection (Greedy FS) was included as a benchmark method. Furthermore, in LASSO, AMP-*L*_0_, and SCAD, the penalization coefficient of the regularization term used is denoted with λ > 0 and thus these methods can be called λ-penalized shrinkage methods. We performed cross-validation (CV) to assess generalization ability, supported by bootstrapping of the data and subsequent re-fitting of the models to assess robustness of the selected features.

In addition to the model accuracy, we evaluated the cost-efficiency of the models as a function of feature measurement costs obtained from actual clinical laboratory measurement kit reference costs from the Helsinki University Hospital. We evaluated the model performance of the methods OSCAR, LASSO, APM-*L*_0_, SCAD, and Greedy FS with respect to the costs calculated with the corresponding number of predictors. This gave us an approximation of the Pareto-front aiming at a good compromise between minimal real-life cost and maximal accuracy, since the underlying problem can be seen as a multi-objective optimization problem of these two objectives.

We also investigated which features were selected as robust predictors. More specifically, we performed bootstrapping (BS), in which the model was fitted 100 times to calculate how often (%) each feature was selected as a predictor when a certain cardinality was set. This enabled us to interpret which features are the most robust predictors that are not sensitive to slight perturbations in the input data.

### Prognostic prediction for advanced prostate cancer in real-world hospital registry data

**Fig 3:**
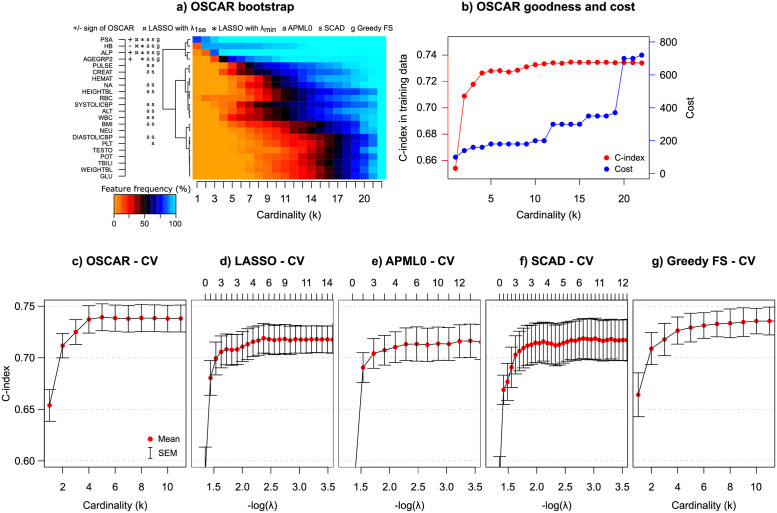
TYKS data: a) OSCAR BS performance. +/- denotes the sign of the coefficient in the model. Positive coefficient: higher predictor value leads towards high risk. Negative coefficient: higher predictor value leads towards low risk. ¤denotes features selected by LASSO with λ_1*se*_ and * denotes features selected by LASSO with λ_*min*_, a denotes features selected by APM-*L*_0_, s denotes features selected by SCAD, g denotes features selected by Greedy FS. Color denotes how often among 100 bootstrap runs a feature is selected when a certain cardinality is set (1 meaning 100%). b) OSCAR accuracy in the TYKS training data (C-index), and cost with respect to the allowed number of predictors. Cost is calculated by kits and a kit price is added if any feature from a kit is used. c) CV performance of OSCAR. d) CV performance of LASSO. The numbers at top indicate the number of predictors selected by a specific λ. e) CV performance of APM-*L*_0_. f) CV performance of SCAD. g) CV performance of Greedy FS. The red dots denote the mean values and the error bars denote the standard errors of the mean (SEM) calculated over the CV folds.

Based on the BS evaluation of the OSCAR method in the TYKS cohort (Fig 3a), PSA was clearly the most robust predictor for the overall survival in prostate cancer. However, as can be seen from Fig 3b and 3c, the original model and CV C-index improved substantially when at least four predictors were chosen. Based on the BS results, the most promising predictors within the explored cardinality values were PSA, hemoglobin (HB), alkaline phosphatase (ALP) and age group (AGEGRP). Notably, the cost remained low when these four predictors were chosen (Fig 3b blue). Adding more predictors did not dramatically improve the OSCAR method accuracy in the training dataset. However, when more predictors were introduced, creatinine (CREAT) and pulse (PULSE) were additionally chosen for prognostic modelling by OSCAR.

In general, OSCAR resulted in improved performance in terms of C-index in CV, when benchmarked against LASSO, APM-*L*_0_, and SCAD methods (Fig 3c–3e). Greedy FS achieved almost the same C-index level than OSCAR, but it required more features for the top-performance. All the methods exhibited roughly similar amount of variation over the CV folds. Of note, OSCAR estimates do not shrink toward zero, but are instead either included or excluded, which may partly explain the saturation effect in the CV performance curves. In this modelling task, the number of predictors (*p* = 22) was relatively low in comparison to the number of patients (*n* = 590). All the methods selected similar predictors (Fig 3a). For example, LASSO with conservative λ (λ_1*se*_) selected three predictors (PSA, HB, and ALP), which are the same as the most important predictors of OSCAR based on the BS. The penalization coefficient λ in LASSO, APM-*L*_0_, and SCAD is typically chosen either based on a local optimum for CV performance (λ_*min*_), or when a solution is within a range of the standard error of the local optimum (λ_1*se*_). In OSCAR, to avoid arbitrary choices for the crucial model penalization, we leverage the use of bootstrapping-based inference to explore feature robustness in addition to the CV generalization ability.

**Fig 4:**
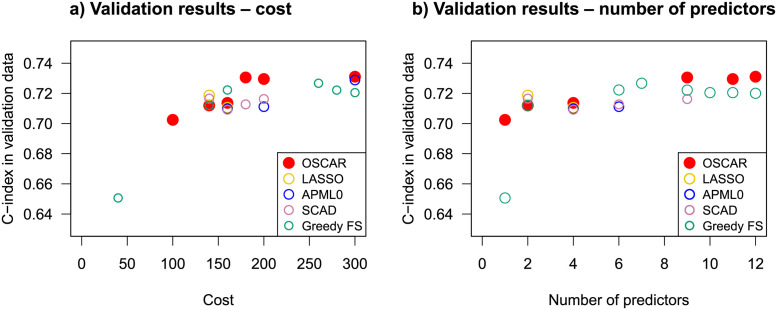
Model accuracy in the validation data cohort for OSCAR (red filled circles), LASSO (yellow hollow circles), APM-*L*_0_ (blue hollow circles), SCAD (light purple hollow circles), and Greedy FS (green hollow circles). a) with respect to the corresponding costs, b) with respect to the corresponding number of predictors. Only the performance of the models in the corresponding approximated Pareto-fronts are presented.

To compare the methods in terms of implementation costs, we investigated the mean C-index in CV of the methods OSCAR, LASSO, APM-*L*_0_, SCAD, and Greedy FS with respect to the costs calculated with the corresponding number of predictors or the values of λ (Fig B in S1 Text). Interestingly, the Pareto-front for OSCAR CV performance vs. cost suggested multiple candidate models, which could be then refined using the domain-expert clinical guidance. The models from these approximated Pareto-fronts were subsequently selected for testing in the left-out validation data to further assess model generalization ability beyond the already observed training data. The observed C-index in the validation dataset (Fig 4) was similar to that in the training dataset. All the methods performed well in the validation dataset, with OSCAR slightly better for low and high costs (or number of predictors).

We further considered scenarios in which the Pareto-front is of no special interest and only a single model prediction is required. For this purpose, the maximum C-index in CV was identified, and the cardinality was selected from this solution within the range of the standard error of the mean (SEM). This is similar approach to those used in LASSO, APM-*L*_0_, and SCAD, but we used SEM instead of standard error. For Greedy FS, the model was selected similarly to OSCAR. Using this strategy, four predictors (PSA, HB, ALP, and AGEGRP Fig 3a) were selected, suggesting a similar model as previously identified with BS. These results demonstrate that even though the methods had a trend toward the same features, the generalization ability of OSCAR was similarly good or better than those using shrinkage-based coefficient estimates.

#### Prediction with kit structure

While the most typical approach is to choose features one at a time, as presented above, features may be available as groups. In clinical practice, features are often measured together as kits (e.g. complete blood count), and therefore including a single feature from a kit in the model leads to availability of measurements for the rest of the kit’s features as well. As the extra features become available at the same cost, it is economical to consider including all of the kit’s features in the model simultaneously.

**Fig 5:**
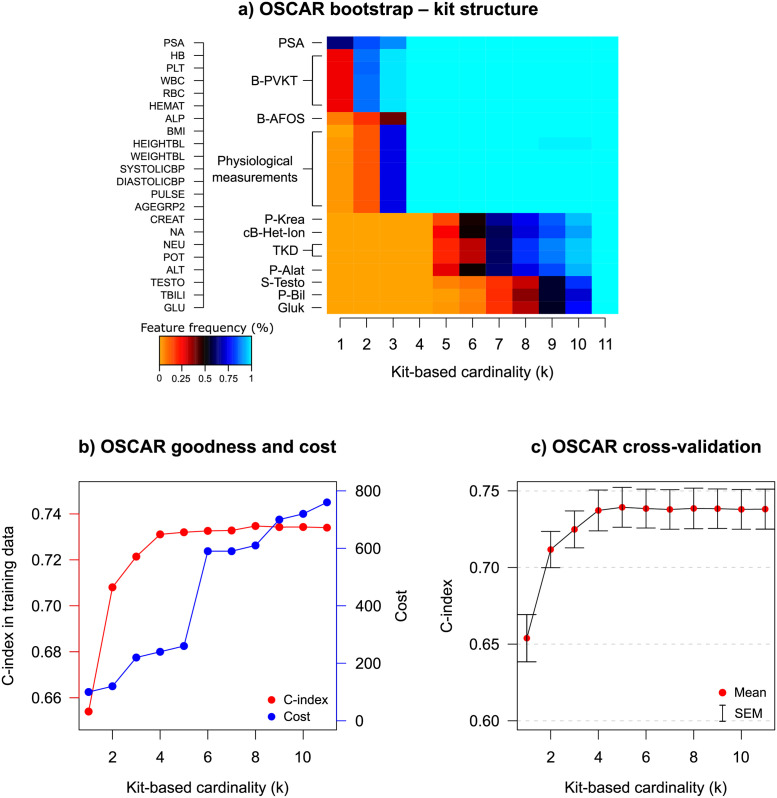
Model performance of OSCAR when the kit structure is used. a) BS performance, b) Goodness (C-index) and cost, c) CV performance. The red dots denote the mean values and the error bars denote the standard errors of the mean (SEM) calculated over the CV folds.

Such a kit structure can be easily included in the OSCAR method (see section 1 in S1 Text), and was investigated in the TYKS dataset. Kit structures used in the analysis are presented in Table 1. Consistent with the non-kit version in the previous subsection, PSA was the most relevant predictor in the TYKS data (Fig 5a). When two kits are allowed, the model suggests B-PVKT (complete blood count), which includes HB, platelets (PLT), white blood cells (WBC), red blood cells (RBC), and hematocrit (HEMAT). While the inclusion of B-PVKT was largely driven by HB, which had been identified as an important predictor in the non-kit approach, four other predictors were now available for model fitting as well. The model fit C-index levels were slightly lower than those with the non-kit prediction. For example, with two kits (total of six predictors), C-index was 0.708 (Fig 5b), whereas the non-kit model of six predictors had C-index on 0.728 (Fig 3b). This is due to trend of including less prognostic features when a kit includes also a highly prognostic feature. However, the cost of six predictors in the non-kit model was 180, whereas the cost for six predictors (two kits) in the kit structure model was 120.

The overall levels of C-index in the CV were similar with or without the kit structure (Fig 5c), when compared to the non-kit prediction (Fig 3c). With the kit structure, the model included features that would not be likely picked by the non-kit model, such as the above mentioned PLT, WBC, and RBC. Furthermore, additional features could be included while keeping the cost low. For example, with two kits the cost was 120 when including six features, whereas without the kit structure, a higher cost was paid with only two features. However, with more features, the risk of overfitting increases. These results demonstrate how the OSCAR method enables the inclusion of clinically relevant kit structures and the addition of multiple model predictors at a given cardinality. In the presented application, the models retained a similar level of generalization ability regardless whether or not the kit structure was taken into account.

### Prognostic prediction for prostate cancer patients in clinical trial data

**Fig 6:**
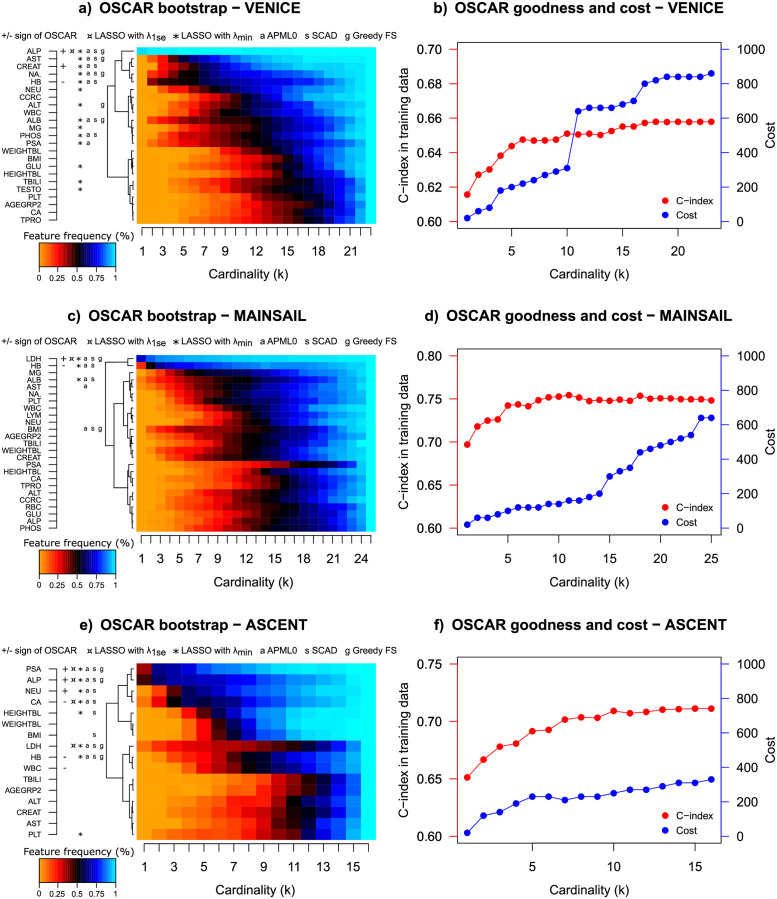
Left panel: BS performance for three trial cohorts. Right panel: Model goodness (C-index) and costs with respect to the allowed number of predictors. Cost is calculated by kits and a kit price is added if any feature from a kit is used.

To investigate how the developed methodology would perform in a more systematically collected and homogenized clinical cohort, we investigate the model performance in three clinical trial data cohorts. One of the striking differences was, that in contrast to the real world cohort TYKS, PSA was significantly less frequently selected as prognostic factor in the three trial data cohorts. In the ASCENT cohort, PSA was distinguished as a prominent predictor (Fig 6 bottom row); however, if only one predictor was allowed, ALP was selected most often in the BS analysis. Furthermore, ALP was selected as the main predictor in the VENICE cohort (Fig 6 top row). In the MAINSAIL cohort, ALP was not detected as a prognostic feature (Fig 6 middle row), and instead, lactate dehydrogenase (LDH) was the most prominent predictor. In the VENICE and MAINSAIL cohorts, HB was selected most often as the second predictor.

ALP and HB were also highly prognostic in the real-world TYKS cohort. Unfortunately, the otherwise highly interesting LDH was not available in the TYKS cohort, due to high percentage of missing values (>80%, Table A in S1 Text). Similarly, TYKS data was missing aspartate aminotransferase (AST), which had notable prognostic power in the VENICE cohort. We note that AST was also, along with LDH, ALP, and HB, detected as one of the most important predictors in the original DREAM 9.5 Prostate Cancer Prediction Challenge [6]. The lack of PSA as the clear top-predictor is also in line with the DREAM 9.5 challenge results, since rather multiple predictors and their interactions need to be considered for maximal prognostic accuracy. Furthermore, the elevated prominence of PSA as a prognostic predictor may be also biased by data generation and reporting, as it is routinely measured in prostate cancer follow-ups, while real-world clinical applications may be less prone to adapt novel markers into routine use.

**Fig 7:**
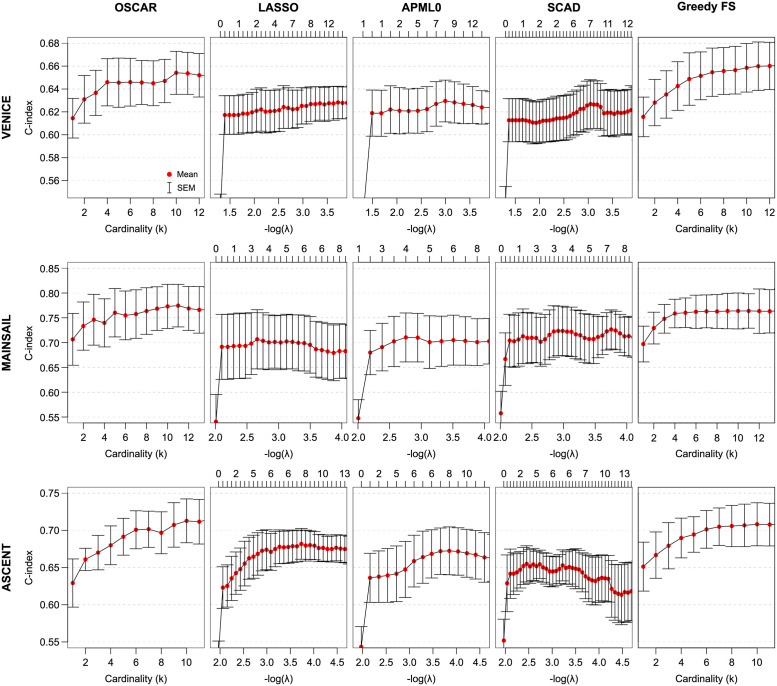
First panel: CV performance of OSCAR in the three trial cohorts. Second panel: CV performance of LASSO in the three trial cohorts. Third panel: CV performance of APM-*L*_0_ in the three trial cohorts. Fourth panel: CV performance of SCAD in the three trial cohorts. Fifth panel: CV performance of Greedy FS in the three trial cohorts. The red dots denote the mean values and the error bars denote the standard errors of the mean (SEM) calculated over the CV folds.

In the VENICE cohort, after the selection of these main predictors that appeared in all the trial cohorts, it became less clear which additional features had the most prognostic power on patient survival. However, based on the model accuracy and the CV results (Figs 6 and 7 top rows), a higher model accuracy was reached with additional predictors. Potential candidate features that improved the model performance were AST, CREAT, sodium (NA), HB, and albumin (ALB). Based on the cardinality within the range of SEM from the maximum C-index in CV, OSCAR selected three predictors (ALP, HB, and CREAT). In the CV analysis, the OSCAR method resulted in a higher mean C-index than LASSO, APM-*L*_0_, and SCAD (Fig 7 top row). Greedy FS resulted in a similar accuracy, further implying the difference between cardinality based methods (OSCAR and Greedy FS) and shrinkage-based methods (LASSO, APM-*L*_0_, and SCAD). However, all the methods suggested similar predictors, indicating their importance and robustness.

In the MAINSAIL cohort, a relatively high C-index was reached by using roughly five predictors, and adding more predictors did not considerably increase the C-index. In the CV, a local maximum was also reached with three predictors (Fig 7 middle row). Thus, based on the BS analysis, in addition to LDH and HB, features like magnesium (MG), body mass index (BMI), ALB, AST, and weight (WEIGHT) were suggested as potential candidates. Based on the cardinality within the range of SEM from the maximum C-index in CV, OSCAR selected two predictors (LDH and HB). When compared to LASSO, APM-*L*_0_, and SCAD, OSCAR again resulted in a higher mean C-index (Fig 7 middle row). As in the VENICE cohort, Greedy FS resulted in a similar level of accuracy in the MAINSAIL cohort.

In the ASCENT cohort, PSA and ALP were the most important predictors (Fig 6 bottom row). Allowing more predictors, such as neutrophils (NEU), calcium (CA), LDH, and HB, further increased the C-index. Based on the cardinality within the range of SEM from the maximum C-index in CV, OSCAR selected six predictors (PSA, NEU, ALP, CA, HB, and WBC). Similar to the other clinical trial cohorts, OSCAR resulted in the highest mean C-index in the CV analysis of the ASCENT cohort when compared to LASSO, APM-*L*_0_, and SCAD (Fig 7 bottom row).

**Fig 8:**
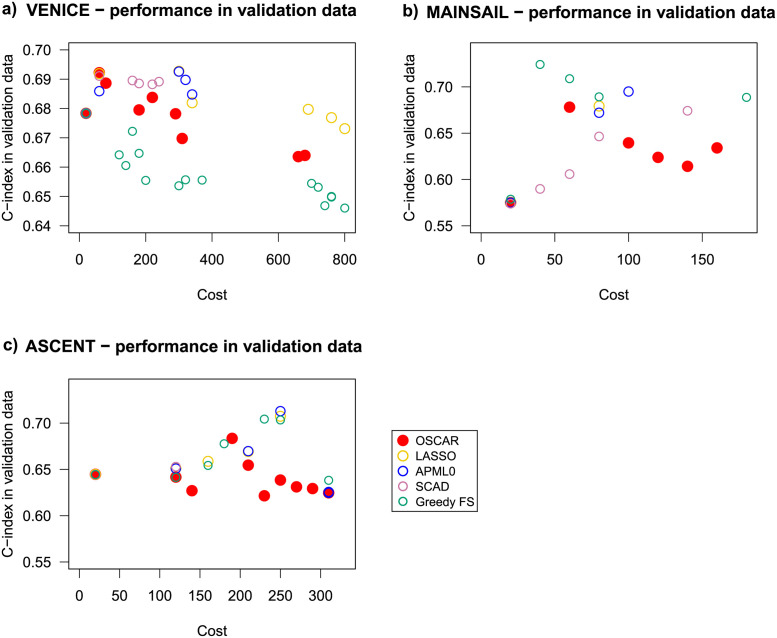
Model accuracy in the validation data cohort for OSCAR (red filled circle), LASSO (yellow hollow circles), APM-*L*_0_ (blue hollow circles), SCAD (light purple hollow circles), and Greedy FS (green hollow circles) in the three trial data cohorts VENICE, MAINSAIL, and ASCENT. Only the performance of the models in the corresponding approximated Pareto-fronts are presented (see Fig C in S1 Text).

To investigate the implementation costs, the mean CV accuracies were inspected with respect to the cost in all three trial data cohorts and for all the methods (OSCAR, LASSO, APM-*L*_0_, SCAD, and Greedy FS) (Fig C in S1 Text). For each of the cohort-method pairings, the approximated Pareto-fronts were analysed. Similarly to the TYKS dataset, OSCAR method resulted in higher accuracies when compared to LASSO, APM-*L*_0_, and SCAD at the same cost levels. Greedy FS resulted in similar levels of accuracies and costs. Next, the models corresponding to the approximated Pareto-fronts were applied in the validation dataset (Fig 8). In the validation data, the models may have exhibited some overfitting as the highest validation C-index was often reached already with a relatively low feature cost. In general, OSCAR performed well in validation considering the goal of simultaneously maintaining a high validation C-index and a low cost. None of the comparison methods excelled over the trial cohorts.

These results demonstrate that the models based on the trial cohorts slightly differ in terms of the selected model parameters from each other, and also from the real-life cohort TYKS. However, part of the differences may be caused by the lack of data in some of the cohorts (e.g. LDH lacking from TYKS). The comparison methods selected similar predictors within a cohort. In general, the OSCAR method improved the prediction accuracy in training data without increasing the cost when compared to the shrinkage-based methods.

### Benchmarking in high-dimensional transcriptomics datasets for prostate cancer recurrence prediction

As high-dimensional ‘omics data present key features for prognostic and predictive modelling for patient’s recurrence and survival in most cancers, we conducted further analysis across three prostate cancer gene expression datasets. Firstly, The Cancer Genome Atlas (TCGA) [21] presents one of the most comprehensive openly available multi-’omics data in cancer, and has been widely utilized for discovering novel molecular patterns across multiple cancer types. Here, we utilized the primary prostate cancer samples (PRAD) with biochemical recurrence status from follow-up, characterized with RNA-seq and processed by the UCSC XenaBrowser platform. Secondly, Taylor et al. [22] provided hundreds of publicly available prostate cancer samples from Memorial Sloan-Kettering Cancer Center (MSKCC) with HuEx-1_0-st exon array transcriptomics profiles for over a hundred primary tumors along with biochemical recurrence information with follow-up times (GEO accession ID GSE21032). Thirdly, Sun et al. [23] profiled 79 patients using HG-U133A Affymetrix microarrays (GEO accession ID GSE25136), with roughly even recurrent and non-recurrent cases, which were used for binarized recurrence status prediction, as follow-up times were unavailable.

#### Model training and validation

TCGA (*n* = 404, total 76 observed recurrence events without censoring) presented the most comprehensive dataset, thus it was used for training all the five methods. One fourth of the data (*n* = 101, with 18 observed events) was set aside as a held-out TCGA validation cohort, while the remaining primary tumor samples (*n* = 303, with 58 observed events) were used for training the models.

**Fig 9:**
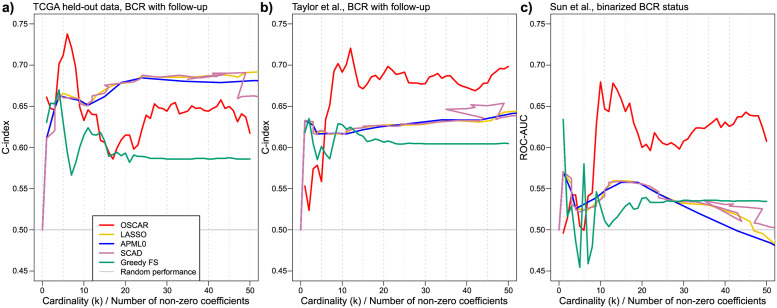
Performance in the high-dimensional transcriptomics data based on model fitting on the TCGA training data. Shrinkage based methods (LASSO, APM-*L*_0_, and SCAD) were plotted as a function of λ; of these, SCAD was the only method for which the number of incorporated features appeared as a non-monotonous function of λ. a) One fourth of the TCGA data (*n* = 101) was left out as a held-out validation dataset. b) Taylor et al. (*n* = 131) with biochemical recurrence with follow-up times was used as an independent validation dataset. c) Sun et al. (*n* = 79) with binarized biochemical recurrence without follow-up times was used as an independent validation dataset.

Results for the recurrence predictions with the five methods are presented in Fig 9. The shrinkage based methods (LASSO, APM-*L*_0_, and SCAD) were plotted as a function of λ; of these, SCAD exhibited a non-monotonous number of non-zero coefficients as a function of the penalization coefficient λ. Of note, all the shrinkage methods were closely clustered together, with only minor differences observed between the methods. For the cardinality-based methods (OSCAR and Greedy FS), a spectrum of model fits from *k* = 1 to *k* = 50 was inspected, and the results of the shrinkage methods were mapped to the cardinality based scale on their count of non-zero coefficients as a function of penalization coefficient λ.

The TCGA held-out dataset was in general well modeled across the spectrum of model complexity, with all models starting with relatively high C-index of 0.65 when using the first few features (Fig 9a). After this, the next few key genes differentiated the cardinality-based methods OSCAR and Greedy FS, with OSCAR choosing a few advantageous features while Greedy FS decreased its C-index to around 0.6, where it remained until *k* = 50. The shrinkage methods performed best after *k* > 10, reaching a C-index of approximately 0.7 as more features were introduced into the models. At higher cardinalities, OSCAR performed slightly worse than the shrinkage methods, while maintaining a reasonable C-index of slightly below 0.65.

The Taylor et al. transcriptomic dataset differentiated OSCAR from the other methods when trained in the TCGA training dataset (Fig 9b); while OSCAR did not initially perform well with a few genes, by *k* = 10 it outperformed the other methods and maintained a C-index of around 0.70. The shrinkage-based methods again performed very similarly, with C-indices ranging between 0.60 and 0.65. Greedy FS maintained a stable performance of C-index of around 0.60 across the cardinality range.

Sun et al. clearly represented the most difficult prediction task for the TCGA-trained models, possibly because it was the oldest dataset, had the lowest sample size, and it lacked follow-up information; the end-point was a binarized recurrence outcome, with ROC-AUC used as the performance metric (Fig 9c). All the methods had initially high variability until *k* = 10, performing only slightly better than random (C-index = 0.5) at best. Between *k* = 10 to *k* = 20 OSCAR and the shrinkage methods improved in performance, with OSCAR gaining an advantage over the other methods with ROC-AUC reaching above 0.65. This was slightly reduced when more features were introduced into the model, but OSCAR remained above ROC-AUC > 0.6.

#### Method comparisons

**Fig 10:**
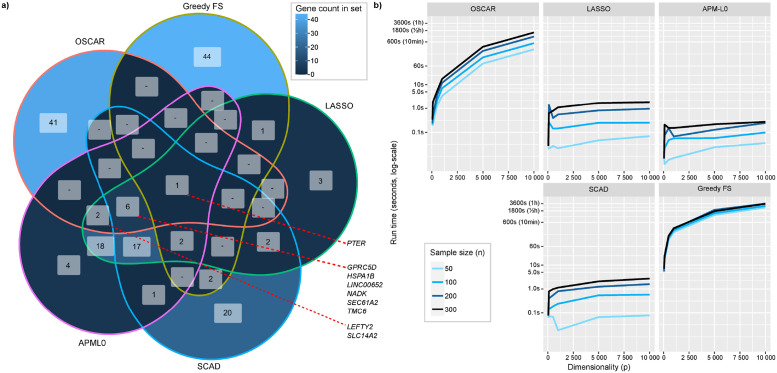
High-dimensional transcriptomics data comparisons in the TCGA dataset. a) Venn diagram for top 50 genes identified by the five feature selection methods. b) Run times on a standard personal desktop subsampled from TCGA across the methods by varying dimensionality (samples from all genes, *p*), and sample size (subsets from all primary tumor samples, *n*).

In addition to validation performance, we observed that the methods separated into roughly two distinct classes. The first class was constituted by those methods that rely on a λ-penalized shrinkage (LASSO, APM-*L*_0_, and SCAD), which formed a very closely performing model across all three validation datasets as seen in Fig 9 panels a-c. The other distinct class was formed of OSCAR and Greedy FS, which instead relied on choosing a suitable cardinality. In order to examine whether this was also reflected on the chosen variables, we chose the top variable candidates up to *k* = 50 for OSCAR and Greedy FS, while for the λ-penalized methods we chose the lowest λ with the first model with 50 non-zero coefficients. A Venn diagram of these variables is presented in Fig 10a and the largest overlap of chosen variables was between LASSO and APM-*L*_0_. However, SCAD somewhat deviated from the other shrinkage-based methods, as it identified 20 genes unique to this method, in contrast to unique gene counts specific to LASSO and APM-*L*_0_ of three and four, respectively. OSCAR and Greedy FS identified notably more unique genes, that were specific to each cardinality-based method.

Regarding the identified top predictors themselves, only one predictor, *PTER*, was detected across all five methods. Further, genes such as *GPRC5D*, *HSPA1B*, *LINC00652*, *NADK*, *SEC61A2*, and *TMC6* were common for four methods including OSCAR, and *LEFTY2* and *SLC14A2* were common for OSCAR, LASSO, and APM*L*_0_. Biological interpretation for the mentioned genes remains challenging and should not be accepted at face-value, for example, for clinical risk predictor use; while perhaps robust modelling-wise, there is no clear connection to the mechanics of prostate cancer recurrence. As presented in the original work in [21, 22], the mechanics underlying prostate cancer progression and recurrence are multi-faceted, and it is likely that associated biological processes would possibly be better explained via copy-number alterations, gene fusions, mutations, or gene expression pathways rather than expression of individual genes. As such, while the model built with OSCAR generalized well across these three validation datasets, its biological applicability ought to be subjected to the scrutiny of the particular expert domain, ideally complemented by other ‘omics.

#### Computational burden of OSCAR in high-dimensional transcriptomics data

While many computationally efficient regularized regression methods rely on fast algorithms, such as coordinate descent-based approaches, our optimization approach utilizes a DC decomposition of the objective function and sophisticated nonsmooth optimization algorithms for finding optimal solutions. However, this inevitably comes at an increased computational cost.

As we performed computational burden simulations on the TCGA and Taylor et al. datasets for Cox models in high-dimensional datasets (*p* = 19 353 and *n* = 404, and *p* = 17 410 and *n* = 140, respectively), we observed that the computational time increased linearly for identifying *k*_*max*_ top features as the desired maximum cardinality *k* was increased (Fig D panel a in S1 Text for TCGA and Fig E panel a in S1 Text for Taylor et al.). Interestingly, as we used the acceleration procedure in OSCAR (see section 2 in S1 Text for details) containing a tuning parameter *γ* for the used percentage of starting points, this parameter was counter-intuitive since it did not provide better solutions as a larger quantity of starting points was provided with a higher value of *γ* (Figs D and E panels b-c in S1 Text). This highlights that designing suitable heuristics for the optimization of an NP-hard nonconvex problem remains a non-trivial task. Lastly, we observed a non-linear increase in the computational time as dimensionality *p* increased (Figs D and E panels d in S1 Text); despite such non-linear increase, the computational burden of OSCAR remained within the scope of a standard personal desktop for even high-dimensional transcriptomics data.

As expected, OSCAR was more computationally intensive than the shrinkage-based methods LASSO, APM-*L*_0_, and SCAD. While OSCAR was computationally more demanding than the shrinkage methods, it retained a computational time lesser than that of Greedy FS across tested parameter grid of dimensionality *p* ∈ {50, 100, 500, 1 000, 5 000, 10 000} and sample sizes *n* ∈ {50, 100, 200, 300} as seen in Fig 10b. These run time simulations were performed by sampling from the TCGA transcriptomics dataset.

## Discussion

In this work, we have introduced a new *L*_0_-regularized regression methodology OSCAR, and demonstrated its use in one hospital registry dataset (TYKS), three datasets from clinical trials (VENICE, MAINSAIL, and ASCENT) and three high-dimensional transcriptomics datasets (TCGA, Taylor et al., Sun et al.). The OSCAR method utilizes the *L*_0_-pseudonorm as a penalty term to restrict the number of predictors. Unlike previous approaches trying to tackle the difficult discrete nature of the *L*_0_-pseudonorm, OSCAR restructures the problem so that no approximation is required and the original solution can be then obtained in an exact manner. Since the pseudonorm is discontinuous and nonconvex, the optimization problem becomes NP-hard and computationally heavy [11]. In the OSCAR method, the *L*_0_-pseudonorm based penalty was rewritten for easier management, and this leads to a regularization term in the form of a DC (Difference of two Convex functions) composition. The optimization was done using DBDC algorithm [17, 18]. This method is more sophisticated and more suitable for nonconvex problems than, for example, the classical coordinate descent. DBDC was also supplemented by a more computationally efficient optimizer LMBM, available in the OSCAR R-package.

We compared OSCAR to LASSO (a widely used method in survival prediction), APM-*L*_0_ (a *L*_0_-based survival prediction method) [15], SCAD-penalized regression [25], and greedy forward selection. All shrinkage-based methods selected similar predictors. In general, OSCAR provided robust and accurate predictions based on the CV analyses, and did not perform significantly worse than the other methods in any of the datasets. This is partly because the *L*_0_-pseudonorm allows the model coefficients to vary freely from zero, unlike, for example, in LASSO, which pushes the coefficients towards zero. LASSO, APM-*L*_0_, and SCAD utilize the coordinate descent in optimization, which is more prone to local optima when compared to the DBDC and LMBM algorithms. Despite the *L*_0_-approach, APM-*L*_0_ performed similarly to LASSO, most likely because it incorporates both *L*_1_ and *L*_2_.

In the TYKS cohort, the OSCAR method suggests PSA, HB, ALP, and age group as the main predictors. Similar trend was also observed if the kit structure was included. PSA reflects the disease severity, especially at disseminated state and in treatment-resistant disease [40]. Thus, PSA has often been acknowledged as an important predictor for prostate cancer, and it is being used in practice to determine and monitor the state or occurrence of prostate cancer. The elevated prominence of PSA as a prognostic predictor in our hospital registry data may thus be biased by data generation and reporting. A high level of ALP is associated with metastases in advanced prostate cancer and it is also measured in the clinical practice to monitor the spreading of cancer into the bones [41]. Metastases typically lead to decreased survival times and, thus, an increased risk of death. Therefore predictors associated with metastases have an intuitive explanation as to why they have prognostic power. HB is generally a good indicator of a person’s health. Similarly to HB, age group is linked to the overall health of a person as overall disease burden is typically higher when physical performance status is lower. Since we predict overall survival, higher age leads to decreased survival time regardless of the cancer related characteristics, which somewhat complicates its survival interpretation.

In the VENICE cohort, ALP prevailed as the most prominent predictor, and AST, CREAT, NA, HB, and ALB followed as additional predictor candidates. As mentioned above, ALP is associated with metastases and thus poor prognosis. AST tests for liver damage, and it has been associated with multiple cancers including prostate, bladder, testicular and small cell lung cancer [42–46]. CREAT is related to kidney malfunction, and NA metabolism also mainly reflects kidney function. Taken together, these prognostic factors therefore reflect potential organ failure or organ damage burden. As such, their use in prognostic models are highly justified and intuitive.

Albumin is a protein that maintains fluid balance and osmolality in bloodstream and it is associated with malnutrition and problems in intake of nutrients in the gut [47]. Compromised intake of nutrients may be caused by cancer, cancer-related decrease in daily performance or cancer treatments, suggesting a potential link between ALB and cancer prognosis [48, 49]. In addition, ALB is considered to reflect liver function and in metastasized, castration-resistant prostate cancer, and lowered levels of ALB is known to associate with increased tumor burden [50, 51].

In the MAINSAIL cohort, HB and ALB were again identified as key prognostic features. In addition, LDH was selected systematically in the BS analysis as a key predictor. LDH is an enzyme participating in energy production in nearly all tissues. Damaged tissues release LDH, which has been linked to cancer burden [48].

Similarly to the VENICE cohort, AST was among the top predictors in the MAINSAIL cohort. In addition, BMI, MG, and WEIGHT had considerable prognostic power, of which MG is a pivotal part of metabolism.

In the ASCENT cohort, similar features were selected consistently in the BS analysis: PSA and ALP, along with NEU, CA, LDH, and HB. NEU are white blood cells that kill bacteria and help in wound healing. They have also been associated with cancer, despite the previous belief of neutrality against cancer [52, 53]. Especially advanced cancer accumulates NEU, which therefore becomes a predictor of poor survival. Unlike in other two trial cohorts, CA was selected among six top predictors in the ASCENT cohort. CA is a mineral especially involved in bone metabolism. Since prostate cancer is prone to metastasize in bones, the CA balance may be related to the cancer development. However, another causation could also be considered since high calcium intake has been associated with increased risk of advanced prostate cancer [54, 55].

Interestingly, when the OSCAR method as well as the four benchmarking methods were applied to a higher dimension recurrence prediction task in the three transcriptomics datasets, the performance of the methods started to split into roughly two main categories. OSCAR and Greedy FS formed a varying bundle of cardinality-based methods, in which addition of new features shifted the model performance metric rather considerably. In contrast, LASSO, APM-*L*_0_ (despite embedding the *L*_0_-pseudonorm), and SCAD penalized models performed almost identically (Fig 9 panels a-c), indicating that their regularization mechanics perform very similarly in real-data applications, despite some differences in the underlying theoretical formulation. In these application examples, this illustrates that while a variety of regularized regression methods exist, some are variants of existing methods, and fundamentally differing approaches are yet to be explored.

The high-dimensional data provided by TCGA [21] and Taylor et al. [22] likely present a more indolent disease phenotype than those of CRPC in the real-world hospital cohort (TYKS) or the three mCRPC clinical cohorts (ASCENT, MAINSAIL, and VENICE). Thus, while we applied here survival modelling for right-censored biochemical recurrence, it is likely that the underlying characteristics for the studies for prostate cancer greatly differ in their molecular processes. This should be noted when interpreting results presented here-in. Further, data was modeled on a very different level (gene expression vs. clinical variables), and ideally, multiple levels of ‘omics would be incorporated together with key clinical characteristics. As such, comprehensive modelling of prostate cancer would leverage such multi-modal data, which imposes its own unique challenges, and remains out of the scope of this methodology-orientated work.

As a future work, it would be interesting to compare OSCAR with more sophisticated Sequential Feature Selection (SFA) algorithms that incorporate forward selection, backward elimination, and their adaptive mixture techniques [56]. It is possible that OSCAR has some beneficial properties due to the inherent nature of the *L*_0_-pseudonorm, while multiple variants of SFA methods have long existed in the feature selection domain. This presents a venue for future research to explore additional properties of the under-studied *L*_0_-pseudonorm in practical applications. While it may be intuitive to first compare the *L*_0_-approach to *L*_1_- and *L*_2_-norm based regularization, more similar counterparts may be found from such SFA algorithms, where norms are not used in the method formulation.

The novelty in practical implementations of the *L*_0_-pseudonorm is highly interesting in the light of our recent application of the OSCAR methodology in the DREAM Anti-PD1 Prediction Challenge. In this crowd-sourced data analysis challenge, the Cox model produced by OSCAR was a top-performer for predicting nivolumab efficacy in non-small cell lung cancer among competing models submitted by over 50 data modelling teams across the world, representing with a diverse set of modelling algorithms ranging from tree-based regression, regularized regression (*L*_1_ and *L*_2_), to deep learning algorithms. This highlights that there is untapped potential in methods, such as the *L*_0_-regularized regression, in various applications where it can outperform competing traditional methods. [57]

Taken together, interesting potential improvements remain, despite the already promising validation results with comparable accuracy and reasonable model features. Due to the inclusion of the *L*_0_-pseudonorm, the optimization problem becomes NP-hard and computationally heavy as illustrated in the high-dimensional transcriptomics data (Fig D in S1 Text for TCGA and Fig E in S1 Text for Taylor et al. Cox model computational burden simulations) and in Fig 9b. Thus, further development of the optimization process, such as using different optimization algorithms or refining the selection of starting points, could potentially improve the running time and the model solutions. Another alternative in the future work is to design a completely new and computationally lean optimizer tailored for the nonconvex and nonsmooth model resulting from the usage of the *L*_0_-pseudonorm and complement it with suitable heuristics. Another potential development is to reformulate the objective function to take into account also the user-provided costs of features and kits. However, this will lead to an even more difficult discrete optimization problem.

## Conclusion

We have developed, tested and made available a novel approach to the *L*_0_-regularized regression, which has previously gone under-represented within the domain of regularized regression, partially due to challenges related to solving the discrete optimization task. Our approach is exact to *L*_0_-penalty as it does not utilize any approximation of the *L*_0_-pseudonorm, but instead uses its exact DC (Difference of two Convex functions) reformulation, bringing the optimization task to the continuous domain. In addition, we have incorporated the kit structure into the method, enabling the selection of grouped features as they are measured in the clinical practice. Since the measurements may have a potentially high costs, the model sparsity allows the selection of the most prognostic features to avoid excessive costs by addition of redundant predictors. The costs were investigated along with model accuracy. This gave us an approximation of the Pareto-front based on the minimal cost and maximal accuracy, since the underlying problem can be seen as a multi-objective optimization problem with two objectives: accuracy and cost. The multi-objective optimization could be regarded as a new way of providing models that are highly relevant to real-world applications, rather than merely optimal according statistical metrics. This way the regularized methodology can also leverage domain-expert knowledge in choosing the final model for real-world applications. Unfortunately, since the transcriptomics data are not collected on a cost-per-gene basis, our Pareto-front approach was not directly applicable to these data. However, simple count of features (i.e. genes in the model) could potentially have applications in designing commercially effective targeted panels using, for example, PCR-based approaches.

The OSCAR method demonstrated efficient and robust performance in the context of metastatic castration resistant prostate cancer in real-world hospital registry data, as well as in three clinical trial data cohorts, and three publicly available high-dimensional transcriptomics data capturing biochemical recurrence. Our results brought insights into the best markers, which to some extent differ between the real-world registry data and the clinical trial data, possibly due to differences in cohort patient characteristics, missingness patterns, or data reporting practices. Furthermore, exploration of the transcriptomics data revealed that the existing shrinkage-based methods are prone to providing similar solutions, despite some incorporating *L*_0_-pseudonorm like characteristics. Thus, we observed that the *L*_0_-pseudonorm may present similar properties as sequential feature selection algorithms rather than the shrinkage-based methods such as LASSO (*L*_1_-norm) or Ridge Regression (*L*_2_-norm). We benchmarked our methodology against highly popular regularization methods, readily available for R users, such as LASSO, and demonstrated comparable or improved performance of our *L*_0_-approach. The OSCAR methodology has been implemented and distributed as a user-friendly R-package, accompanied by a wide range of useful helper functions and a set of efficient Fortran optimizers called from within the R-package. The OSCAR method is easily accessible through the Central R Archive Network (CRAN).

## Supporting information

S1 Text Supplementary information file. The file contains sections: 1) Restricting the number of kits in OSCAR, 2) Acceleration procedure for high-dimensional data, 3) Data characteristics, 4) Correlations, 5) Approximated Pareto-fronts, 6) High-dimensional transcriptomics data.
(PDF)
