# Supplementary material for: OSCAR: Optimal subset cardinality regression using the L0-pseudonorm with applications to prognostic modelling of prostate cancer
Source: PLoS Comput Biol. 2023 Mar 10;19(3):e1010333. doi: 10.1371/journal.pcbi.1010333 (PMC10032505; doi:10.1371/journal.pcbi.1010333)
Supplement: S1 Text — The file contains sections: 1) Restricting the number of kits in OSCAR, 2) Acceleration procedure for high-dimensional data, 3) Data characteristics, 4) Correlations, 5) Approximated Pareto-fronts, 6) High-dimensional transcriptomics data. (PDF) [file pcbi.1010333.s001.pdf]

OSCAR - Supplementary information

Anni S. Halkola[1◑], Kaisa Joki[1◑], Tuomas Mirtti[2,3,4], Marko M. Mäkelä[1], Tero Aittokallio[1,5,6,7], Teemu D. Laajala[1,8*]

1 Department of Mathematics and Statistics, University of Turku, Turku, Finland
2 Research Program in Systems Oncology, Faculty of Medicine, University of Helsinki, Helsinki, Finland
3 Department of Pathology, Diagnostic Center, Helsinki University Hospital, Helsinki, Finland
4 Department of Biomedical Engineering, School of Medicine, Emory University, Atlanta, Georgia, United States of America
5 Institute for Molecular Medicine Finland (FIMM), HiLIFE, University of Helsinki, Helsinki, Finland
6 Institute for Cancer Research, Oslo University Hospital, Oslo, Norway
7 Oslo Centre for Biostatistics and Epidemiology (OCBE), University of Oslo, Oslo, Norway
8 Department of Pharmacology, University of Colorado Anschutz Medical Campus, Aurora, Colorado, United States of America

◑These authors contributed equally to this work.
* Corresponding author: teelaa@utu.fi

1 Restricting the number of kits in OSCAR

In many applications, some predictors are typically always measured together meaning that they belong to the same kit. This is typical, for example, in medicine since many medical tests yield values for more than one predictor. Thus, sometimes instead of restricting the number of nonzero coefficients it may be more useful to limit the number of used kits and allow only the coefficients in the selected kits to be nonzero. In this subsection, we generalize the cardinality constraint to take into account the kit structure.

We assume that in the model we have altogether p single predictors, $k \le p$ different kits and that each predictor belongs to exactly one kit. To incorporate the kit structure, we define a *kit matrix* $\boldsymbol{W} \in \mathbb{R}^{k\times p}$ with elements

$$(\boldsymbol{W})_{i,j} = \begin{cases} 1, & \text{if the predictor } j \text{ belongs to the kit } i \\ 0, & \text{otherwise.} \end{cases}$$

Thus, the kit matrix $\boldsymbol{W}$ is a $(0,1)$-matrix and its row i describes the predictors in the kit i. In addition, we can guarantee that $\sum_{i=1}^{k}(\boldsymbol{W})_{i,j} = 1$ holds for each predictor $j \in \{1,\ldots,p\}$ since a predictor is only included in one kit.

Remark 1. *Sometimes a predictor may belong to more than one kit (i.e. if $\sum_{i=1}^{k}(\boldsymbol{W})_{i,j} > 1$ for some j) and this type of a situation violates the assumption that a predictor is only included in one kit. However, the problem can always be modified such a way that this assumption holds. In the modification, we first calculate for each predictor j the number of kits k_j where it belongs. If $k_j > 1$ we divide the original coefficient β_j linked to the predictor j in the model (e.g. the scaled log partial likelihood) to k_j parts by writing $\beta_j = \beta_{j,1} + \ldots + \beta_{j,k_j}$. Due to this, we have altogether*

$p' = \sum_{i=1}^{k}\sum_{j=1}^{p}(\boldsymbol{W})_{i,j}$ predictors in the final optimization problem. In addition, each kit containing the coefficient β_j of the original predictor j is linked to exactly one of the coefficients $\beta_{j,i}$, $i = 1,\dots,k_j$. This enables us to write the original matrix $\boldsymbol{W} \in \mathbb{R}^{k\times p}$ as $\boldsymbol{W}' \in \mathbb{R}^{k\times p'}$ where each column has only one nonzero element.

Next, we define a mapping $\boldsymbol{g} : \mathbb{R}^p \times \mathbb{R}^{k\times p} \to \mathbb{R}^k$ with the formula

$$g_i(\boldsymbol{\beta}, \boldsymbol{W}) := \sum_{j=1}^{p}(\boldsymbol{W})_{i,j}|\beta_j| \quad \text{for all } i = 1,\dots,k,$$

where $\boldsymbol{\beta} \in \mathbb{R}^p$ and $\boldsymbol{W} \in \mathbb{R}^{k\times p}$. For the used kit matrix, the result of this mapping is a vector containing the sums of the absolute values of the coefficients belonging to each kit.

Example 1. *Assume that we consider a problem with six predictors (i.e. $\boldsymbol{\beta} \in \mathbb{R}^6$). In addition, we have three kits and the kit matrix is of the form*

$$\boldsymbol{W} = \begin{pmatrix} 1 & 1 & 1 & 0 & 0 & 0 \\ 0 & 0 & 0 & 1 & 1 & 0 \\ 0 & 0 & 0 & 0 & 0 & 1 \end{pmatrix}.$$

Thus, for example, predictors $j = 1, 2, 3$ are available in the same kit (e.g. hematocrite, hemoglobin, and red blood cell count). Similarly predictors $j = 4$ and $j = 5$ belong to the same kit and the predictor $j = 6$ is measurable alone. For this selection, we obtain

$$\boldsymbol{g}(\boldsymbol{\beta}, \boldsymbol{W}) = \left(|\beta_1| + |\beta_2| + |\beta_3|,\, |\beta_4| + |\beta_5|,\, |\beta_6|\right)^\top.$$

If we decide to restrict the number of kits to be $K \in \{1,\dots,k\}$, then the cardinality constraint can be written as

$$\|\boldsymbol{g}(\boldsymbol{\beta}, \boldsymbol{W})\|_0 \le K.$$

Similarly to the case with single predictors (see subsection "Restricting the number of model features"), we can use the largest-k norm for $\boldsymbol{g}(\boldsymbol{\beta}, \boldsymbol{W})$ to rewrite the cardinality constraint in an equivalent form

$$\|\boldsymbol{g}(\boldsymbol{\beta}, \boldsymbol{W})\|_1 - |||\boldsymbol{g}(\boldsymbol{\beta}, \boldsymbol{W})|||_{[K]} = 0.$$

Since each predictor $j \in \{1,\dots,p\}$ belongs to exactly one kit we have

$$\|\boldsymbol{g}(\boldsymbol{\beta}, \boldsymbol{W})\|_1 = \|\boldsymbol{\beta}\|_1.$$

Thus, for the selected number of kits $K \in \{1,\dots,k\}$ this yields us the cardinality-constrained problem (see (5) in the main paper) of the form

$$\begin{cases} \min\limits_{\boldsymbol{\beta}\in\mathbb{R}^p} & -l(\boldsymbol{\beta}) \\ \text{s.t.} & \|\boldsymbol{\beta}\|_1 - |||\boldsymbol{g}(\boldsymbol{\beta}, \boldsymbol{W})|||_{[K]} = 0, \end{cases} \tag{S1}$$

where l is the scaled log partial likelihood (see (2) in the main paper). It is worth to notice, that the problem for single predictors is a special case of (S1). It can be obtained by selecting the kit matrix $\boldsymbol{W}$ coinciding with the $p \times p$ identity matrix $\boldsymbol{I}$ meaning that each kit contains only one predictor. To solve the problem (S1), we again use the penalty function approach [1,2] allowing us to rewrite (S1) as an unconstrained minimization problem

$$\min_{\boldsymbol{\beta}\in\mathbb{R}^p} \quad f(\boldsymbol{\beta}) = -l(\boldsymbol{\beta}) + \rho\left(\|\boldsymbol{\beta}\|_1 - |||\boldsymbol{g}(\boldsymbol{\beta}, \boldsymbol{W})|||_{[K]}\right), \tag{S2}$$

where $\rho > 0$ is a positive penalization parameter. The objective f is still DC (Difference of two Convex functions) and the DC presentation $f = f^1 - f^2$ is easily constructed by selecting the convex functions

$$f^1(\boldsymbol{\beta}) = -l(\boldsymbol{\beta}) + \rho\|\boldsymbol{\beta}\|_1 \quad \text{and} \quad f^2(\boldsymbol{\beta}) = \rho|||\boldsymbol{g}(\boldsymbol{\beta}, \boldsymbol{W})|||_{[K]}.$$

Due to this, DBDC can also be utilized to solve the scaled log partial likelihood with the kit structure. An interesting feature of the kit penalized reformulation (S2) is that it can also be seen as a modification of the l_1-penalty since the only difference is the largest-k norm term $-\rho|||\boldsymbol{g}(\boldsymbol{\beta}, \boldsymbol{W})|||_{[K]}$. Note that this is the term restricting and controlling the upper bound for the number of nonzero kits in the problem.

For the kit structure, OSCAR is presented in Algorithm A. Note that Step 1 generates starting points based on the kit structure. Otherwise the algorithm is really similar to the one with single predictors (see Algorithm 1 in the main article) and with the selection $\boldsymbol{W} = \boldsymbol{I}$ the execution is identical. In addition, when we solve the scaled log partial likelihood allowing only K_{max} kits to be used, we obtain as a by-product a solution also for each cardinality-constrained problem with a smaller number of used kits. It is also possible to execute OSCAR without fixing beforehand the number of kits by setting $K_{max} = k$. In this case, the method provides a solution for each possible number of kits.

2 Acceleration procedure for high-dimensional data

In high-dimensional data, the number of features p is larger than the number of observations n. Next, we introduce a new acceleration procedure used to boost calculations, in particular, in high-dimensional data. It is needed because in high-dimensional data, the previously described heuristic to generate initial points is not the best possible one. The reason for this is that the more high-dimensional the data is, the more time-consuming it is to solve the problem (S1) presented in the previous section. Thus, it may require a lot of computational time to solve the high-dimensional problem (S1) from each of the starting points during each iteration of OSCAR. Furthermore, in high-dimensional data most of the features are irrelevant and do not contain any useful information. Due to this, all of the features or kits should not be used to construct the starting points or be included in the problem (S1).

For the above mentioned reason, we have boosted calculations in high-dimensional data by presenting an acceleration procedure utilizing a low-dimensional convex subproblem and a reduced-sized version of the original problem (S1). The low-dimensional subproblem is fast to be solved and it is used to spot the most promising features or kits together with starting points linked to them. After the most promising features or kits are spotted we generate based on them the reduced-sized version of the original problem (S1) and solve it from the obtained starting points. Since we do not solve the original high-dimensional problem (S1) but its reduced-sized version we are able to significantly accelerate the computations. In addition, the user can affect the size of the reduced problem and how many starting points are used. More detailed description of the acceleration procedure is given below. Since the procedure is presented for the problem (S1) with kit structure the case with single predictors is its special case.

Assume that we are starting the execution of the iteration $K+1$ of the OSCAR method. Thus, we have already obtained a solution $\boldsymbol{\beta}_K^*$ for the problem (S1) with K kits (if $K = 0$ then $\boldsymbol{\beta}_0^* = \mathbf{0}$). In addition, we know that $K < k \leq p$, where k is the maximum number of the kits and p is the maximum number of single predictors. First, we collect to the sets C_K and F_K the indices of the kits $\{1, 2, \ldots, k\}$ and features $\{1, 2, \ldots, p\}$, respectively, already used in the solution vector $\boldsymbol{\beta}_K^*$. The reduced solution vector is denoted by $\boldsymbol{\beta}_{F_K}^* \in \mathbb{R}^{|F_K|}$ and it just contains the predictors of $\boldsymbol{\beta}_K^* \in \mathbb{R}^p$ which are nonzero.

Input: The values of features $\boldsymbol{x}_i$, the survival times y_i, the labels $\delta_i \in \{0,1\}$, the kit matrix $\boldsymbol{W}$ and the maximal number of kits $K_{max} \in \{1,2,\ldots,k\}$ until which the cardinality-constrained problem is solved.

Output: For $K = 1,\ldots,K_{max}$, gives the solution $\boldsymbol{\beta}^*_K$ for the cardinality-constrained problem with K kits.

Step 0: (*Initialization*) Solve the scaled log partial likelihood model without any regularization (see (3) in the main article) with DBDC or LMBM and denote the solution by $\bar{\boldsymbol{\beta}}$. Set $\boldsymbol{\beta}^*_0 = \mathbf{0}$ and $K = 1$.

Step 1: (*Starting points*) For the cardinality-constrained problem with K kits, initialize the set of starting points $S_K = \emptyset$. For $j = 1,\ldots,k$ construct the point $\boldsymbol{\beta}^j_0$ with the formula

$$\beta^j_{0,l} = (\boldsymbol{W})_{j,l}\bar{\beta}_l + (1-(\boldsymbol{W})_{j,l})\beta^*_{K-1,l} \quad \text{for } l = 1,\ldots,p.$$

and if $\|\boldsymbol{g}(\boldsymbol{\beta}^j_0, \boldsymbol{W})\|_0 > K-1$ then add the point to the set S_K.

Step 2: (*Penalty function problem*) Do the following steps A–C for all $\boldsymbol{\beta}^j_0 \in S_K$ to obtain solutions $\boldsymbol{\beta}^*_{K,j}$

Step A: Select a positive initial value for the penalization parameter ρ.

Step B: Solve the problem (S2) with the DBDC or LMBM method starting from $\boldsymbol{\beta}^j_0$ and denote the solution with $\hat{\boldsymbol{\beta}}_j$.

Step C: If $\|\boldsymbol{g}(\hat{\boldsymbol{\beta}}_j, \boldsymbol{W})\|_0 = K$, then set $\boldsymbol{\beta}^*_{K,j} = \hat{\boldsymbol{\beta}}_j$. Otherwise increase the value of the penalization parameter ρ and go to Step B.

Step 3: (*Solution*) Select the best solution $\boldsymbol{\beta}^*_K$ for the cardinality-constrained problem (S1) with K kits using the formula

$$\boldsymbol{\beta}^*_K = \arg\min_j \{ -l(\boldsymbol{\beta}^*_{K,j}) \}.$$

Update $K = K+1$. If $K \le K_{max}$, then go to Step 1. Otherwise go to Step 4.

Step 4: Return $\boldsymbol{\beta}^*_K$ for all $K = 1,\ldots,K_{max}$.

Algorithm A: OSCAR with the kit structure

In order to find promising starting points during the iteration $K+1$, the idea is to take the previous solution $\boldsymbol{\beta}^*_K$ as a base and test one by one how each missing kit would improve it. Note however, that we are only optimizing the values of the predictors in the added kit. Therefore, the nonzero predictors of $\boldsymbol{\beta}^*_K$ are not changed and their values are treated as constants (this corresponds to the vector $\boldsymbol{\beta}^*_{F_K}$). Due to this, we look through each kit h from the set $\{1,\ldots,k\} \setminus C_K$. After the kit h is selected we first define for it the set F_h containing predictors in the kit and the corresponding solution vector $\boldsymbol{\beta}_{F_h} \in \mathbb{R}^{|F_h|}$. The value for the predictor vector $\boldsymbol{\beta}_{F_h}$ is obtained by solving the low-dimensional convex subproblem

$$\min_{\boldsymbol{\beta}_{F_h} \in \mathbb{R}^{|F_h|}} \quad f(\boldsymbol{\beta}_{F_h}) = -\frac{2}{n}\sum_{i=1}^{m}\left\{\sum_{j\in D_i}\left[a_j + \left(\boldsymbol{x}^{F_h}_j\right)^\top \boldsymbol{\beta}_{F_h}\right] - d_i \ln\left(\sum_{j\in R_i} e^{\left[a_j + \left(\boldsymbol{x}^{F_h}_j\right)^\top \boldsymbol{\beta}_{F_h}\right]}\right)\right\}, \text{(S3)}$$

where $a_j = (\boldsymbol{x}_j^{F_K})^\top \boldsymbol{\beta}^*_{F_K}$ for $j = 1, \ldots, n$ are the constant values obtained based on the previous solution. In addition, both observation vectors $\boldsymbol{x}_j^{F_K}$ and $\boldsymbol{x}_j^{F_h}$ contain only those features from $\boldsymbol{x}_j$ which are found from the sets F_K and F_h, respectively. Thus, in the subproblem we solve the scaled log partial likelihood (see subsection "Cox's proportional hazards model"), where we fit the kit h among the previously obtained nonzero predictors $\boldsymbol{\beta}^*_{F_K}$.

One nice feature of the subproblem (S3) is that whenever we substitute the predictors F_h of $\boldsymbol{\beta}^*_K$ with values in the solution vector $\boldsymbol{\beta}^*_{F_h}$ we obtain a starting point with $K+1$ kits. Another useful property is that when we select $\hat{\boldsymbol{\beta}}_{F_h} = \mathbf{0}$ the value $f(\hat{\boldsymbol{\beta}}_{F_h})$ coincides with the objective function value obtained with the previous solution $\boldsymbol{\beta}^*_K$ with K kits. Due to this, we can guarantee that a starting point, where features F_h of the solution $\boldsymbol{\beta}^*_K$ are replaced with $\boldsymbol{\beta}^*_{F_h}$, always gives a smaller objective function value for the problem (S1) with $K+1$ kits than the previous solution $\boldsymbol{\beta}^*_K$. Note also that whenever only single predictors are used the convex subproblem (S3) is always one-dimensional.

Therefore, when the acceleration procedure is used Step 1 of Algorithm A is substituted with the following: "First, for each kit $h \in \{1, \ldots, k\} \setminus C_K$ solve the subproblem (S3) and constitute a starting point as previously discussed. Second, select a set of starting points S_K to be used in Step 2 of Algorithm A." Before the selection of the starting points we have $k - K$ different options. However, we do not want to use all of them due to the high-dimensional data. Thus, the user can select a value for the parameter $\gamma \in (0, 1]$, which presents the percent of starting points used and we select only $\lceil \gamma(k-K) \rceil$ of them. The selection is done based on the objective function values obtained for the subproblem (S3), since only the best $\lceil \gamma(k-K) \rceil$ starting points in terms of the objective function values are used.

Finally, when we move on to Step 2 of Algorithm A we first reduce the size of the original problem (S1). This reduced problem contains only those kits which were already used in the previous solution $\boldsymbol{\beta}^*_K$ together with the ones constructing the starting points selected in Step 1. This means that in the reduced version of the problem (S1) we have altogether $K + \lceil \gamma(k-K) \rceil$ kits instead of the original k ones. Thus, the only difference in the reduced version of the problem (S1) is that we just leave out part of the kits together with the predictors which belong to these kits.

3 Data characteristics

Table A. Data characteristics

Predictor	TYKS	MAINSAIL	VENICE	ASCENT
AGEGRP				
≤ 64	41	140	165	79
65-74	201	181	191	159
≥ 75	348	73	92	119
BMI	27.6 ± 4.25 (24.9%)	28.5 ± 5.20 (1.5%)	28.5 ± 5.20 (1.5%)	28.3 ± 4.62 (0.3%)
HEIGHTBL	175.9 ± 6.17 (21.4%)	174.2 ± 7.97 (1.3%)	172.5 ± 7.38 (0%)	175.3 ± 7.50 (0%)
WEIGHTBL	84.9 ± 14.4 (15.8%)	86.0 ± 16.3 (0%)	83.3 ± 14.8 (0%)	87.3 ± 16.7 (0.3%)
SYSTOLICBP	142.8 ± 21.4 (36.6%)	-	-	-
DIASTOLICBP	76.3 ± 13.3 (46.1%)	-	-	-
PULSE	72.4 ± 13.6 (36.8%)	-	-	-
HB	12.8 ± 1.77 (2.5%)	12.5 ± 1.55 (1.8%)	12.6 ± 1.31 (0%)	12.5 ± 1.48 (0.8%)
PLT	237.9 ± 70.8 (2.5%)	295.5 ± 102.3 (1.8%)	261.5 ± 88.4 (0.7%)	263.1 ± 89.7 (1.1%)
WBC	1.89 ± 0.33 (2.5%)	1.95 ± 0.40 (1.8%)	1.92 ± 0.32 (0%)	1.87 ± 0.33 (0.8%)
RBC	4.20 ± 0.57 (2.5%)	4.16 ± 0.48 (1.8%)	-	-
HEMAT	38.4 ± 4.93 (2.5%)	-	-	-
NEU	1.32 ± 0.51 (43.9%)	1.62 ± 0.50 (2.0%)	1.50 ± 0.42 (0.7%)	1.47 ± 0.42 (1.7%)
POT	4.10 ± 0.38 (7.5%)	-	-	-
ALP	4.50 ± 0.69 (8.0%)	5.05 ± 0.87 (0%)	5.12 ± 0.89 (0.4%)	4.91 ± 0.77 (0.6%)
ALT	3.05 ± 0.60 (15.8%)	3.01 ± 0.52 (0.3%)	3.06 ± 0.55 (0.2%)	2.98 ± 0.48 (0.8%)
AST	x	3.25 ± 0.39 (0%)	3.25 ± 0.40 (1.3%)	3.22 ± 0.38 (0.8%)
CA	x	2.30 ± 0.14 (0%)	2.30 ± 0.21 (2.0%)	2.37 ± 0.13 (0.3%)
CREAT	4.52 ± 0.37 (1.9%)	4.39 ± 0.24 (0%)	4.42 ± 0.22 (0.2%)	4.49 ± 0.26 (0%)
LDH	x	5.44 ± 0.44 (0%)	-	5.42 ± 0.46 (3.1%)
PSA	3.01 ± 2.01 (1.2%)	4.46 ± 1.68 (0.8%)	4.51 ± 1.60 (1.1%)	4.24 ± 1.58 (0.3%)
TBILI	2.17 ± 0.54 (33.9%)	1.74 ± 0.44 (0.5%)	2.10 ± 0.44 (0.4%)	1.90 ± 0.44 (3.4%)
TESTO	-0.64 ± 1.02 (46.4%)	x	-1.18 ± 1.69 (2.0%)	x
NA	140.2 ± 3.44 (7.5%)	139.9 ± 3.09 (0%)	139.5 ± 3.57 (0.4%)	-
MG	x	-0.15 ± 0.10 (0%)	-0.12 ± 0.20 (6.0%)	-
PHOS	x	0.05 ± 0.23 (0%)	0.11 ± 0.30 (4.7%)	-
ALB	x	42.9 ± 3.85 (0%)	41.2 ± 4.52 (2.2%)	-
TPRO	x	69.7 ± 5.96 (0%)	72.7 ± 5.91 (4.7%)	-
LYM	x	0.13 ± 0.56 (2.0%)	-	-
CCRC	x	4.52 ± 0.29 (37.8%)	4.57 ± 0.27 (0.4%)	-
GLU	1.90 ± 0.23 (28.1%)	1.94 ± 0.34(0%)	1.84 ± 0.31 (1.6%)	-

Data characteristics for prostate cancer patients in the data cohorts: TYKS, MAINSAIL, VENICE, and ASCENT. The distribution of patients in the age groups is presented. For the other features (see abbreviations in the main text Table 1), mean ± standard deviation is presented, with the percentage of missing values in parentheses. x indicates that the feature is available but over 50% of the values is missing. - indicates that the feature is completely missing from the cohort.

4 Correlations

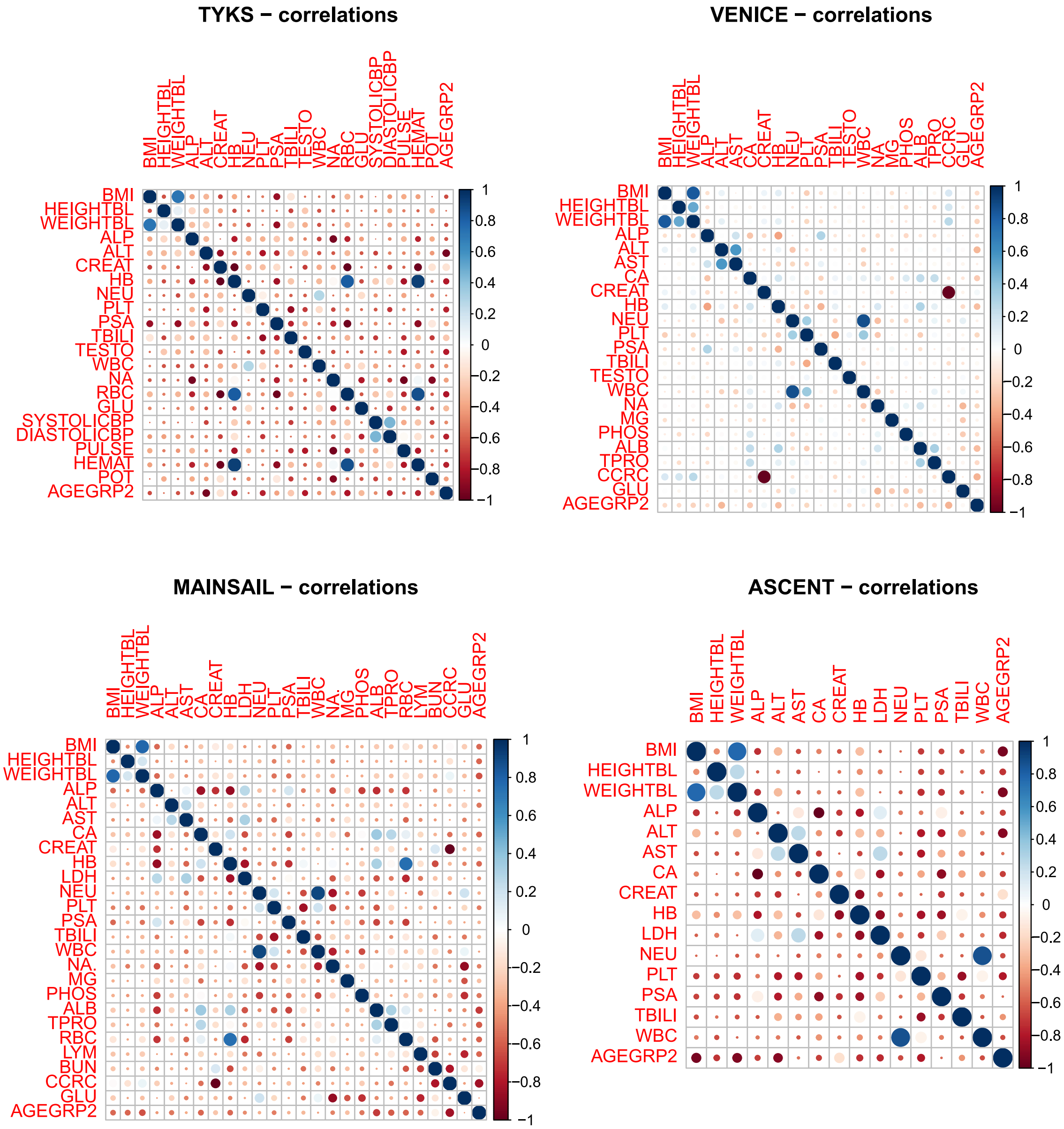

Fig A. Spearman correlations for each data cohort for features with less than 50% of missing values. The correlations were calculated before imputation. See abbreviations in the main text Table 1.

5 Approximated Pareto-fronts

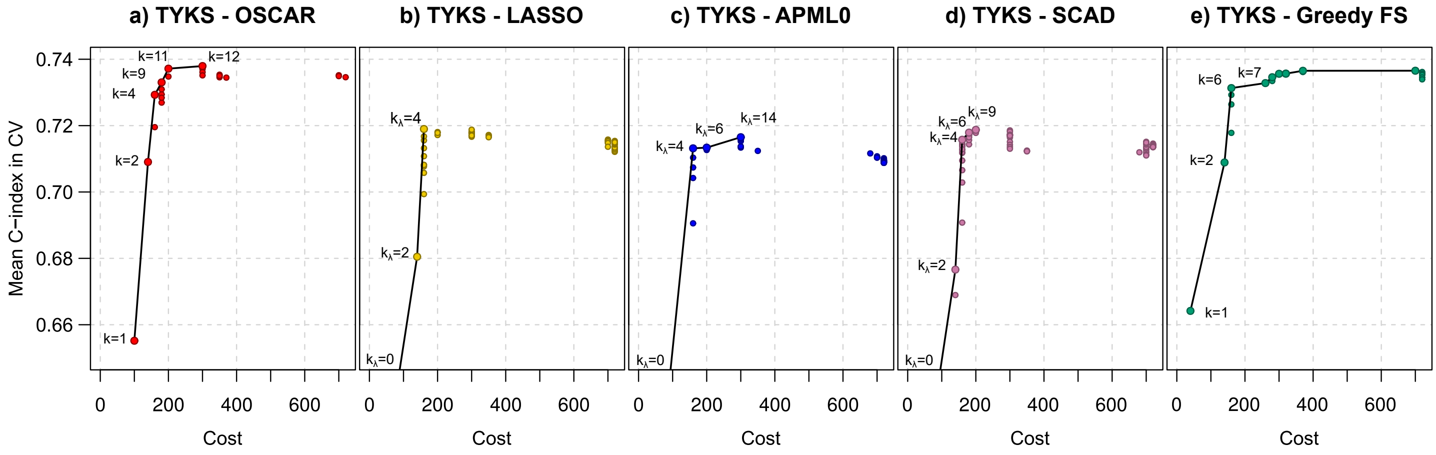

Fig B. Model accuracy in CV with respect to the cost for a) OSCAR, b) LASSO, c) APM-L_0, d) SCAD, and e) Greedy FS. The approximated Pareto-front is marked with black line. Number of predictors in each Pareto-point is noted next to the point. For Greedy FS the remaining Pareto-points have 9, 10, 11, 12, 15, and 16 predictors. The costs were calculated with the corresponding number of predictors (OSCAR and Greedy FS) or corresponding values of λ (LASSO, APM-L_0, and SCAD) using predictors chosen in the model fitted for the entire training data (e.g. Fig 3b in the main article). For each λ, the notation k_λ marks the number of predictors in the model fitted for the entire training data.

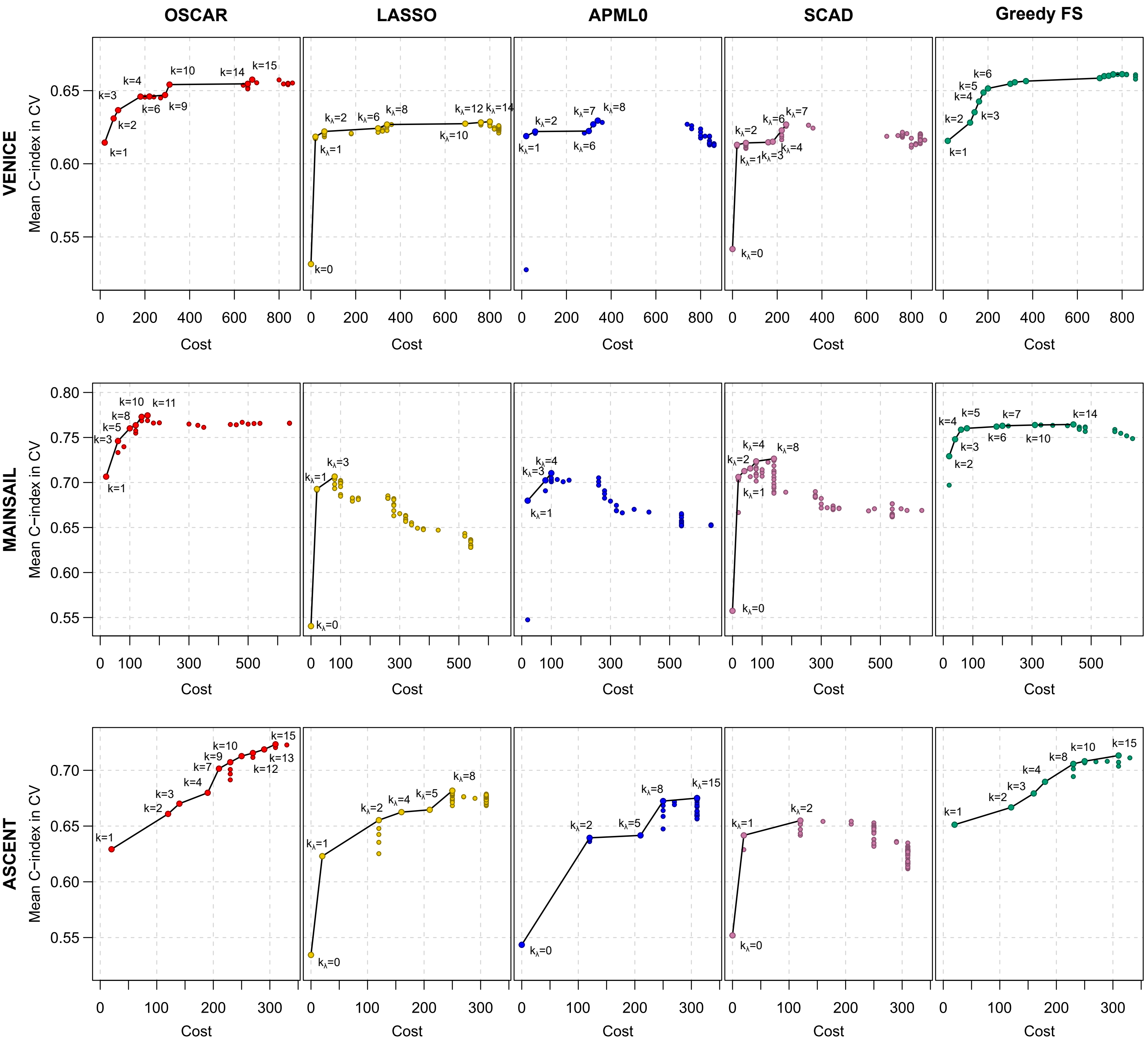

Fig C. Mean prediction accuracy in CV with respect to the cost with the methods OSCAR, LASSO, APM-L_0, SCAD, and Greedy FS for the trial data cohorts VENICE (top row), MAINSAIL (middle row), and ASCENT (bottom row). The approximated Pareto-front is denoted by the black line. For each λ, the notationk_λ marks the number of predictors in the model fitted for the entire training data.

6 High-dimensional transcriptomics data

6.1 Data generation

Three high-dimensional transcriptomics datasets were processed for benchmarking and data analysis:

- The Cancer Genome Atlas (TCGA) [3] (Xena Hub identifier "TCGA-PRAD") dataset with $n = 404$ primary samples and gene expression $p = 19\ 353$.
- Taylor et al. [4] (GEO accession code "GSE21032") with $n = 131$ primary samples and gene expression $p = 17\ 410$.
- Sun et al. [5] (GEO accession code "GSE25136") with $n = 79$ primary samples and gene expression $p = 12\ 784$.

TCGA data was downloaded using the *UCSCXenaTools* R-package [6] (version 1.4.8), while Taylor et al. and Sun et al. were downloaded using the *GEOquery* R-package [7] (version 2.64.2). TCGA gene expression was normalized using RNA-Seq by Expectation Maximization (RSEM) [8], with further log-transformation $log_2(x + 1)$. The downloaded raw CEL files from Taylor et al. and Sun et al. were processed using the *oligo* R-package [9] (version 1.60.0), with Robust Multichip Average (RMA) normalization with background subtraction, quantile normalization and summarization via median-polish as per default parameters. The *biomaRt* R-package [10] (version 2.52.0) was used to extract the latest gene symbol annotations for the expression arrays (Affymetrix Human Exon 1.0 ST Array for Taylor et al. and Affymetrix Human Genome U133A Array for Sun et al.). The *curatedPCaData* R-package [11] (version 0.9.42) was used for harmonizing the input data, along with extracting key clinical metadata such as recurrence information. We limited the transcriptomics data here to just primary tumor samples from patients with non-metastatic disease subtype.

6.2 Time analysis details

For computational burden simulations, sampling was performed from the original data without replacement by taking subsets of the genes (dimensionality p) with a varying sample size n. A grid of cardinality values in $k_{max} \in \{2, 3, 4, 5, 7, 10, 20, 30, 50\}$ was tested together with the acceleration parameter $\gamma \in \{10^{-5}, 10^{-4}, 2 \times 10^{-4}, 5 \times 10^{-4}, 10^{-3}, 5 \times 10^{-3}, 10^{-2}, 2 \times 10^{-2}\}$ (Fig D and E panels a-c). In addition, ten replicates were sampled at $p \in \{50, 100, 200, 500, 1\ 000, 2\ 000, 5\ 000, 10\ 000\}$ (Fig D and E panels d) to evaluate the computational burden as a function of p. The code used for benchmarking *oscar* run times is provided in the GitHub at './data-raw/benchmarks.Rmd'.

6.3 Model validation details

For model validation of the high-dimensional transcriptomics data, the TCGA full dataset was randomly split into 3/4 teaching data and 1/4 held-out validation data. Genes were filtered based on a criterion that over half of the samples were required to have a unique expression value after processing. An intersect of common gene names was taken after this filtering, resulting in a final dimensionality of $p = 10\ 253$ genes. Per each gene, the expression values were z-score transformed to make model coefficients comparable across studies. All models were fit to the teaching dataset of TCGA ($n = 303$) for the common gene symbol intersection.

The latest *oscar* R-package from GitHub was used for all transcriptomics analyses, with the LMBM solver and the acceleration parameter $\gamma = 2 \times 10^{-4}$. For LASSO penalized models, the *glmnet* R-package [12] (version 4.1-4) was used. The *APM-L_0* R-package [13] (version 0.10) with

default parameters and the *ncvreg* R-package [14] (version 3.13.0) with SCAD penalty were used for further benchmarking. The greedy forward selection was implemented using *coxph* from the *survival* R-package [15] (version 3.3-1) and the code is provided in the *oscar* GitHub at ‘./data-raw/greedy.R’. The code used for running model validations is provided at ‘./data-raw/validate.R’.

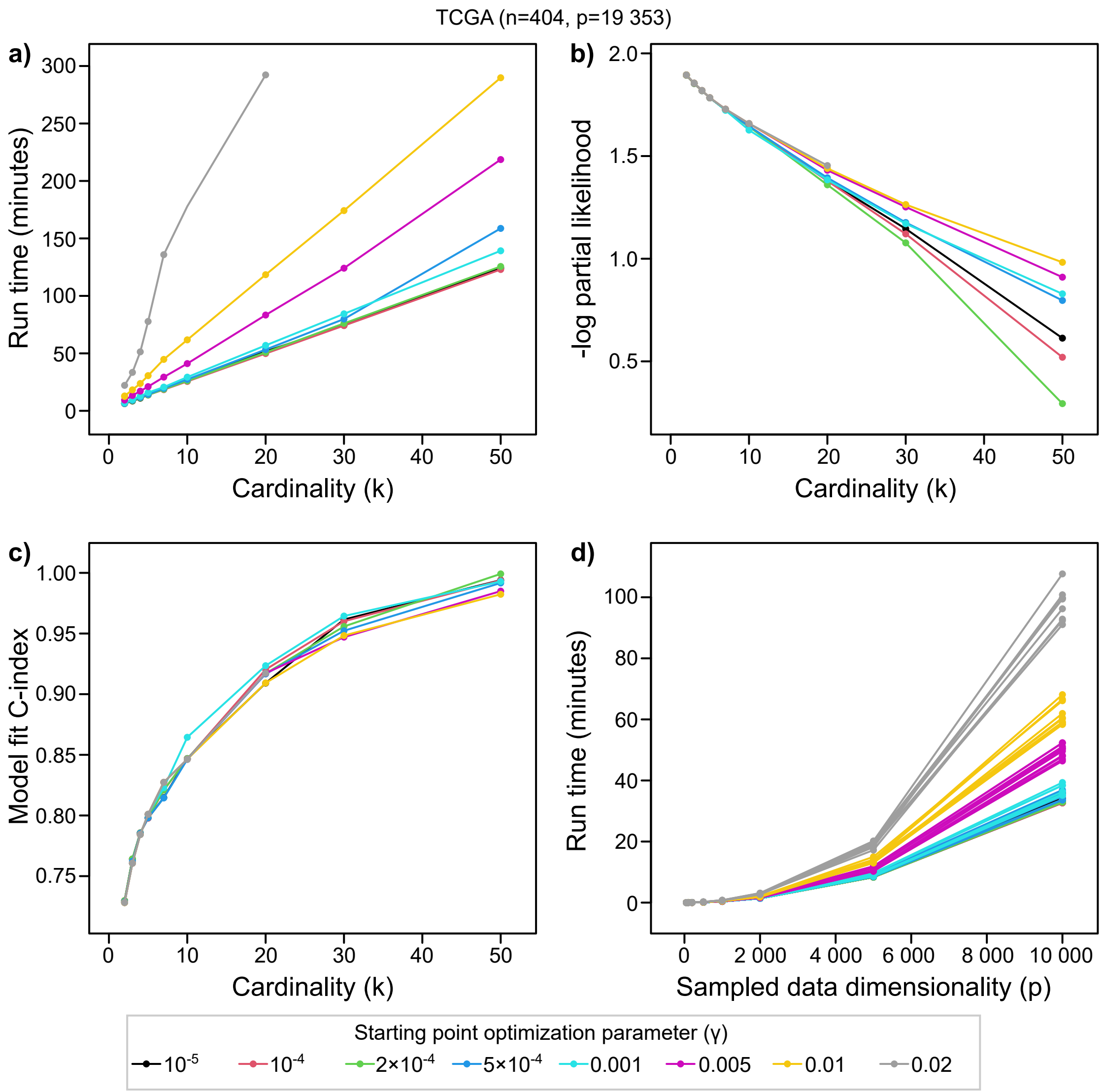

Fig D. Computational burden of the OSCAR methodology in TCGA (full $n = 404$ samples and $p = 19\ 353$ dimensionality): a) Run times in the full TCGA data for different values of the tuning parameter γ as a function of the cardinality k_{max}. b) Model fit log partial likelihood for different values of the tuning parameter γ in the full data. c) Model C-index for different values of the tuning parameter γ in the full data. d) Ten smaller subsets were generated from TCGA with varying p to examine γ as a function of dimensionality.

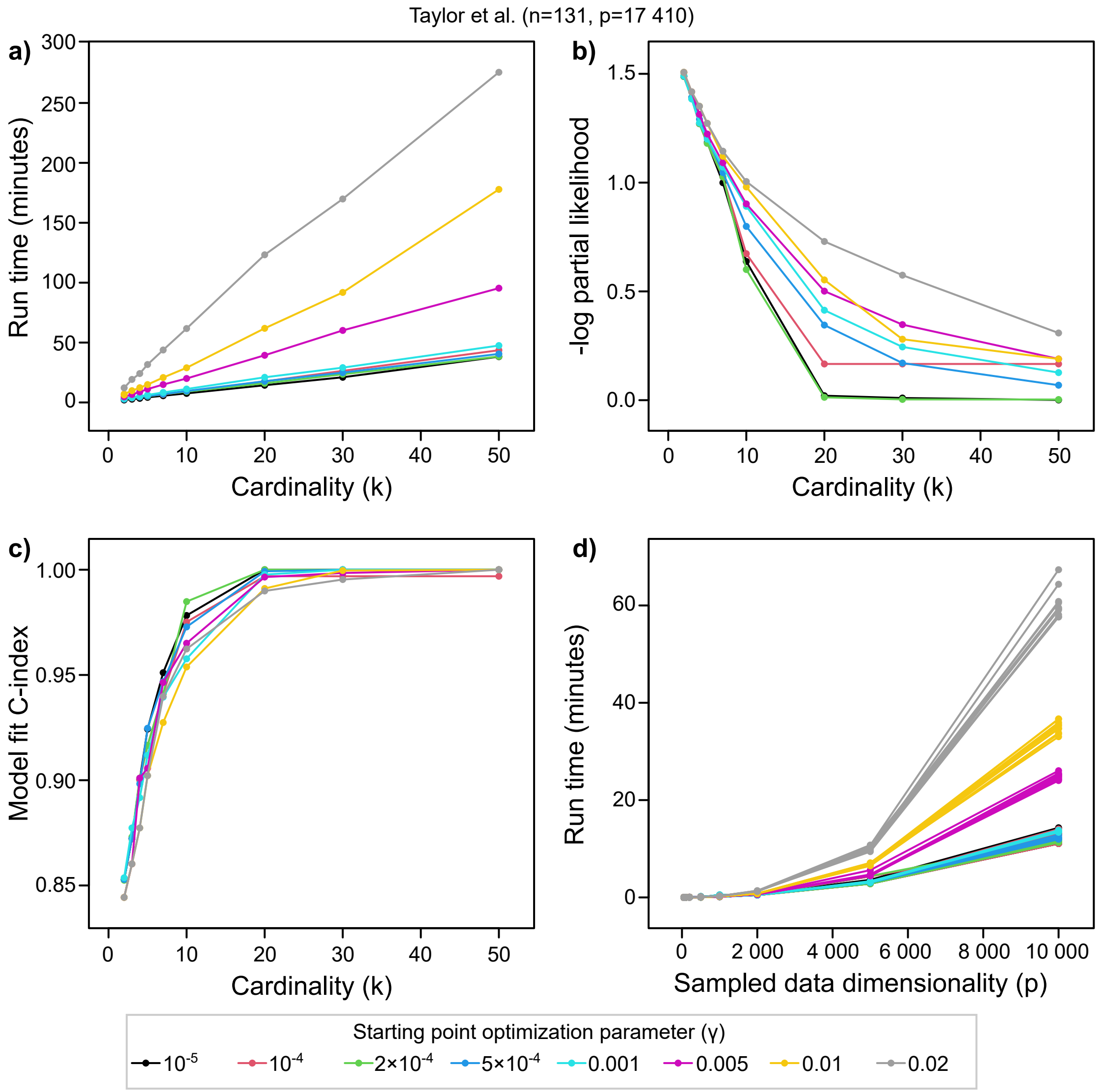

Fig E. Computational burden of the OSCAR methodology in Taylor et al. (full $n = 131$ samples, and $p = 17\ 410$ dimensionality): a) Run times in the full Taylor et al. data for different values of the tuning parameter γ as a function of the cardinality k_{max}. b) Model fit log partial likelihood for different values of the tuning parameter γ in the full data. c) Model C-index for different values of the tuning parameter γ in the full data. d) Ten smaller subsets were generated from Taylor et al. with varying p to examine γ as a function of dimensionality.

References

1. Nocedal J, Wright SJ. Numerical Optimization. Berlin: Springer; 2006.
2. Zangwill WI. Non-linear programming via penalty functions. Management Science. 1967;13(5):344–358. doi:10.1287/mnsc.13.5.344.
3. Cancer Genome Atlas Research Network. The Molecular Taxonomy of Primary Prostate Cancer. Cell. 2015;5(163). doi:10.1016/j.cell.2015.10.025.
4. Taylor BS, Schultz N, Hieronymus H, Gopalan A, Xiao Y, Carver BS, et al. Integrative genomic profiling of human prostate cancer. Cancer Cell. 2010;18(1):11–22. doi:10.1016/j.ccr.2010.05.026.
5. Sun Y, Goodison S. Optimizing molecular signatures for predicting prostate cancer recurrence. Prostate. 2009;69(10):1119–27. doi:10.1002/pros.20961.
6. Wang S, Liu X. The UCSCXenaTools R package: a toolkit for accessing genomics data from UCSC Xena platform, from cancer multi-omics to single-cell RNA-seq. Journal of Open Source Software. 2019;4(40):1627. doi:10.21105/joss.01627.
7. Davis S, Meltzer PS. GEOquery: a bridge between the Gene Expression Omnibus (GEO) and BioConductor. Bioinformatics. 2007;23(14):1846–7. doi:10.1093/bioinformatics/btm254.
8. Li B, Dewey CN. RSEM: accurate transcript quantification from RNA-Seq data with or without a reference genome. BMC Bioinformatics. 2011;12(323). doi:10.1186/1471-2105-12-323.
9. Carvalho BS, Irizarry RA. A Framework for Oligonucleotide Microarray Preprocessing. Bioinformatics. 2010;26(19):2363–7. doi:10.1093/bioinformatics/btq431.
10. Durinck S, Spellman PT, Birney E, Huber W. Mapping identifiers for the integration of genomic datasets with the R/Bioconductor package biomaRt. Nature Protocols. 2009;4(8):1184–91. doi:10.1038/nprot.2009.97.
11. Laajala TD, Sreekanth V, Soupir A, Creed J, Calboli F, Singaravelu K, et al. curatedPCaData: A harmonized resource of integrated prostate cancer clinical, -omic, and signature features. bioRxiv. 2023;doi:10.1101/2023.01.17.524403.
12. Simon N, Friedman J, Hastie T, Tibshirani R. Regularization paths for Cox's proportional hazards model via coordinate descent. Journal of Statistical Software. 2011;39(5):1–13. doi:10.18637/jss.v039.i05.
13. Li X, Xie S, Zeng D, Wang Y. Efficient ℓ_0-norm feature selection based on augmented and penalized minimization. Statistics in Medicine. 2018;37(3):473–386. doi:10.1002/sim.7526.
14. Breheny P, Huang J. Coordinate descent algorithms for nonconvex penalized regression, with applications to biological feature selection. Annals of Applied Statistics. 2011;5(1):232–253.
15. Therneau TM, Grambsch PM. Modeling Survival Data: Extending the Cox Model. New York: Springer; 2000.
